# Gold Quantum Rods: Modulation of Singlet and Triplet Exciton Populations by the Rod Length

**DOI:** 10.1021/jacs.6c10470

**Published:** 2026-08-12

**Authors:** Guiying He, Lianshun Luo, Avirup Sardar, Weijie Ji, Mircea Cotlet, Rongchao Jin

**Affiliations:** † Department of Chemistry, 6612 Carnegie Mellon University, Pittsburgh, Pennsylvania 15213, United States; ‡ Center for Functional Nanomaterials, 8099 Brookhaven National Laboratory, Upton, New York 11973, United States

## Abstract

Gold quantum rods (QRs) of Au_60_, Au_78_, Au_96_, and Au_114_ (protected by thiolate ligands) exhibit exclusive fluorescence, which is different from the shorter Au_42_ QR with dual emission (fluorescence + phosphorescence). Herein, we report the excitation wavelength-dependent near-infrared-II photoluminescence (PL) and exciton dynamics of this series of QRs. Interestingly, the fluorescence quantum yield (QY) of the QRs is much higher (2–10×) when the lowest singlet excited state (S_1_) is excited compared to the excitation of high-lying states (S_n_). A metastable intermediate singlet state (denoted S′) is identified by transient absorption spectroscopy when S_n_ is excited, and this S′ state leads to fast “skybridge” intersystem crossing (ISC), which contributes primarily (>60%) to the total triplet population. With increasing aspect ratio (AR) of QRs (from 6.3 to 18.7), nonradiative processes accelerate, leading to fast decay of the S_1_ state (hence, less QY) and of the T_1_ state (barely phosphorescent). A detailed energy flow mechanism is determined for the QRs after photoexcitation, which offers a design principle for manipulation of exciton dynamics toward potential utilization of higher excited states in applications.

## Graphical abstract

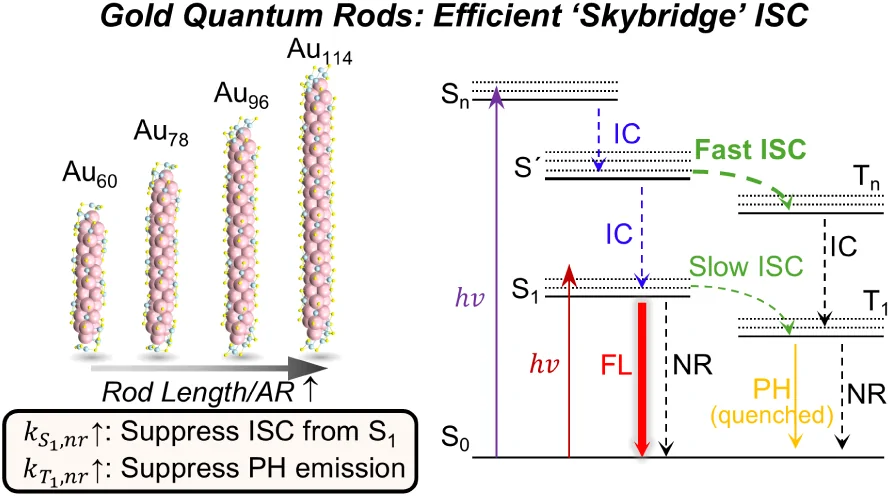

## Introduction

Photoluminescence (PL) in the second near-infrared (NIR-II) region (1000–2500 nm, also called shortwave infrared, SWIR) is of particular importance in a wide range of applications, such as deep-tissue biological imaging,^1^ cancer surgery and therapy,^2^ solar energy utilization,^3,4^ quantum computing, and telecommunications.^5^ With respect to the materials’ development for such applications, atomically precise nanoclusters (APNCs) of metals and alloys have emerged as a new class of NIR-II materials with several advantages, including tunable optical properties by precise structural control, high stability, low toxicity, and ease of preparation via wet chemistry.^6–13^ Generally, the structure of APNCs consists of a metal core and a shell of metal-containing complex-like staple motifs, e.g., the monomeric RS–Au–SR (where −SR = thiolate). Such APNCs possess a quantized electronic structure^6^ and thus show single-electron transitions and molecule-like exciton dynamics.^14–17^ In recent years, APNCs have been increasingly recognized as promising NIR-II luminescent materials with highly efficient triplet population; they exhibit room-temperature phosphorescence (PH) and thermally activated delayed fluorescence (TADF).^3,18–21^ The intersystem crossing (ISC) process from the lowest singlet excited state (S_1_) to the lowest triplet excited state (T_1_) can be manipulated by the structure/size, surface ligands, heterometal doping, and the surrounding environments of APNCs.^22–27^ The high triplet yield has inspired exploration of APNCs as triplet sensitizers for photon upconversion via triplet–triplet annihilation^28,29^ and also as photocatalysts.^30–33^ The observed rapid ISC process (e.g., picoseconds (ps)) suppresses the prompt fluorescence in APNCs compared to other emission processes such as PH and TADF.^25,26^ For instance, in a recent study on Au_52_(SR)_32_, the ISC from the S_1_ state was determined to be ∼11 ps, followed by triplet decay in ∼100 ns.^18,34^ In some cases, time-resolved PL measurements revealed three components, including prompt fluorescence (FL), TADF, and PH.^19,35^ Doping or alloying has been reported to be an effective strategy for PL enhancement.^13,34,36–39^

Recently, a periodic series of gold quantum nanorods (QRs), including Au_42_, Au_60_, Au_78_, Au_96_, and Au_114_ (periodicity: 18 Au atoms) has been developed,^40^ which extends the absorption and emission into the NIR-II region by increasing the aspect ratio (AR) while retaining the same diameter.^41^ These QRs possess a constant diameter of 3.15 Å for the kernel but exhibit increasing lengths from 19.76 to 58.79 Å (i.e., AR from 6.3 to 18.7). Interestingly, the ISC process from S_1_ to T_1_ benefits from a small reorganization energy but is suppressed due to small spin–orbit coupling.^42^ Among the QRs, Au_42_ exhibits NIR dual emission with FL at 875 nm and PH at 1040 nm under either S_1_ excitation (808 nm) or higher singlet excited-state S_n_ excitation (e.g., 400 nm), which was also theoretically simulated by a three-state model (S_0_, S_1_, and T_1_).^42,43^ Experimentally, the PL quantum yield (QY) was determined to be up to 20.9% for Au_60_, with its lifetime of 0.90 ns, hence, fluorescence in nature.^41^ The longer QRs also show single emission, i.e., 1145 nm for Au_60_, 1400 nm for Au_78_, 1620 nm for Au_96_, and 1870 nm for Au_114_.^41,44^ Theoretical analysis^43^ shows that the relevant Au_24_(SR)_20_ (as “seeds” of QRs) should exhibit dual fluorescence, while the elongated ones (Au_42_ and Au_60_) should show FL/PH dual emission. However, the elongated Au QRs (Au_60_, Au_78_, Au_96_, and Au_114_) show single fluorescence emission experimentally,^41^ even though the triplet population was predicted in theoretical calculations.^43^

In this work, by combining photoluminescence studies with transient absorption measurements, we determine the exciton dynamics in the QRs of Au_60_, Au_78_, Au_96_, and Au_114_, including the internal conversion (IC), ISC, radiative, and nonradiative decays of singlet and triplet states. Our results indicate anti-Kasha–Vavilov behavior in QRs, which explains the excitation wavelength-dependent QY and exciton dynamics. An intermediate singlet state (S′) is identified, which contributes to fast ISC to a high triplet state (T_n_) and thus explains the observed interesting photophysical behavior. The excited-state absorption “signature” is observed by transient absorption upon S_n_ excitation. With increasing length of QRs, the normal ISC process (S_1_ → T_1_) is increasingly suppressed; indeed, no triplet state population is observed for Au_96_ and Au_114_ under S_1_ excitation; even for shorter Au_60_ and Au_78_ QRs, their triplet yield is less than 10% under S_1_ excitation. In contrast, under S_n_ excitation, the triplet yield is more than 60%. The nonradiative decays of S_1_ and T_1_ states increase drastically, leading to a drop in QY and the disappearance of PH. A detailed Jablonski diagram is put forth to interpret the complex exciton dynamics for this unique series of QRs.

## Results and Discussion

### Steady-State Optical Properties

The Au QRs were synthesized in solution and purified by thin-layer chromatography according to previous reports.^10,40,41,45^ Electrospray ionization mass spectrometry (ESI-MS) analyses of these four QRs confirmed the purity of the samples (Figure S1). All QRs exhibit a common absorption peak (Figure 1a) at ∼400 nm (nearly constant) and an AR-dependent intense NIR absorption peak. The ∼400 nm absorption band is insensitive to AR, which involves an isotropic exciton according to previous calculations on the Au_42_ QR (which also showed such a peak).^45^ Compared to the absorption peak of 806 nm for Au_42_, the increase in AR dramatically shifts the absorption peak to 1078 nm for Au_60_, 1320 nm for Au_78_, 1540 nm for Au_96_, and 1732 nm for Au_114_. The NIR peak of each QR arises from the strongly polarized HOMO–LUMO transition, with its dipole moment component primarily in the longitudinal direction.^40,43^

**Figure 1:**
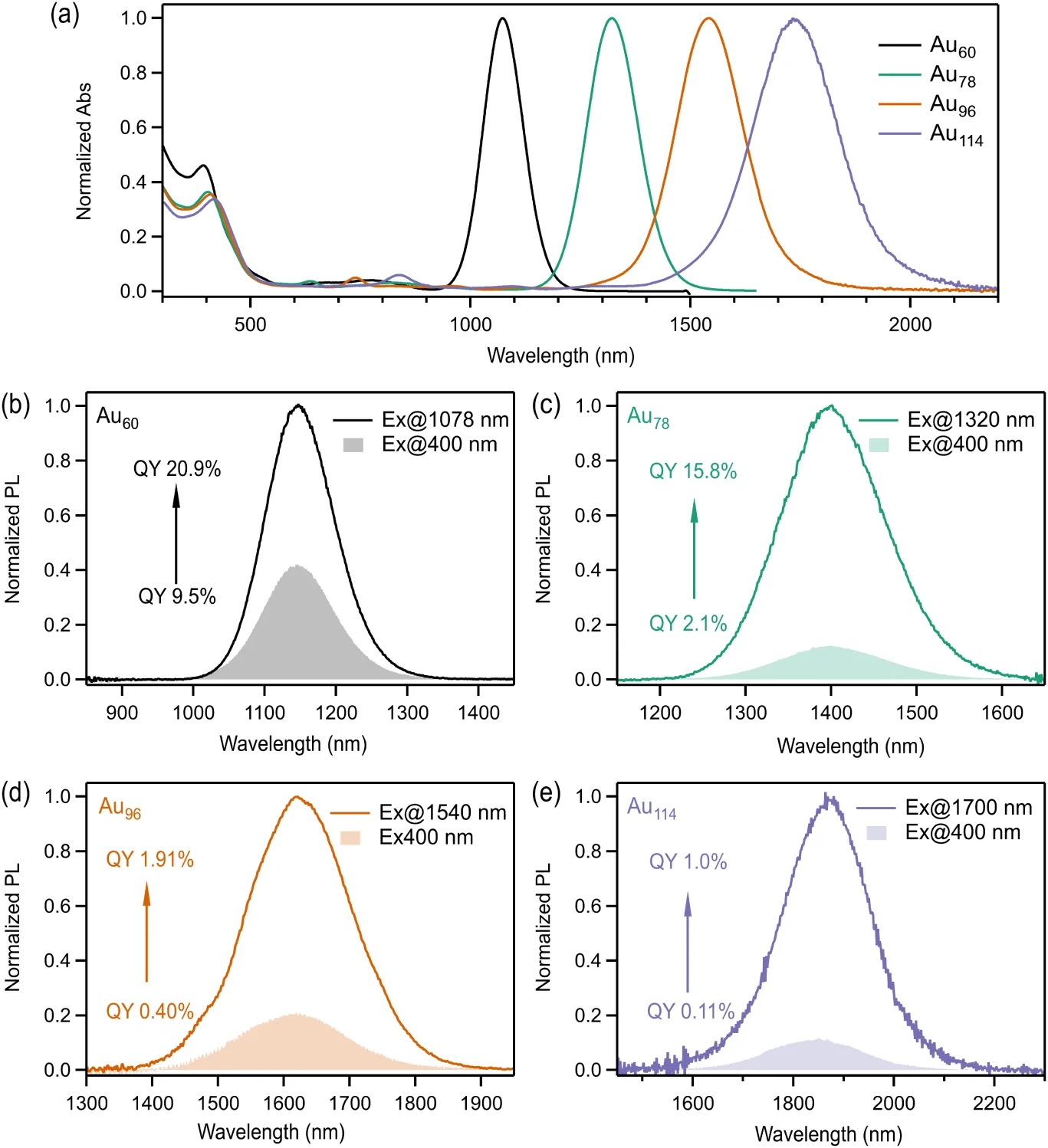
Steady-state absorption of QRs (a) and excitation wavelength-dependent PL spectra of Au_60_ (b), Au_78_ (c), Au_96_ (d), and Au_114_ (e). Solvent: toluene-*d*_8_ (to avoid interference from overtone vibrational peaks of regular toluene).

The small optical gap (*E*_g_) of Au QRs led to photoluminescence in the NIR-II range, with a PL peak at 1145 nm for Au_60_, 1400 nm for Au_78_, 1620 nm for Au_96_, and 1870 nm for Au_114_ (Figure 1b–e).^41^ The QRs longer than Au_42_ exhibit sole fluorescence with no obvious phosphorescence, which is different from the dual emission of Au_42_ (FL at 875 nm and PH at 1040 nm).^10,41,46^ We note that, for Au_60_, very weak phosphorescence (1400–1600 nm) was observed when excited by higher-energy photons (e.g., 400 and 500 nm, Figure S2a and inset); however, no phosphorescence peak can be identified under S_1_ excitation (Figure S2b and inset). The fluorescence spectra are identical (except for the absorbance-normalized peak intensity) regardless of the excitation wavelength, indicating no additional emissive species. The excitation spectrum of Au_60_ (probed at 1145 nm emission) shows a weaker 400 nm peak compared to the absorption spectrum (Figure S3), indicating that the fluorescence of Au_60_ is less contributed by S_n_ excitation than S_1_ excitation. We rationalize that the different excitation wavelengths may have modulated the S_1_ and T_1_ exciton populations in the QRs via anti-Kasha–Vavilov processes;^47–49^ see more discussions below.

The QY of Au_60_ is 20.9% under S_1_ excitation (1078 nm), while it decreases to 9.5% under S_n_ excitation (400 nm), indicating that about half of excitons are lost (Figure 1b). Similarly, for longer QRs, we observe significant exciton loss under S_n_ excitation compared to S_1_ excitation (Figure 1c–e), that is, ∼87% loss for Au_78_, ∼79% for Au_96_, and ∼89% for Au_114_. Correspondingly, a lower QY was observed with increasing length of QRs because of the increasing nonradiative processes of the S_1_ state with a shrinking HOMO–LUMO gap.

### Exciton Dynamics of Au_60_ QRs

To determine the exciton dynamics of QRs, we performed excitation wavelength-dependent femtosecond (fs) and nanosecond (ns) transient absorption (TA) measurements. Considering the absorption and emission extended into NIR-II, we performed TA probing from 850 to 1550 nm to distinguish the signatures of different excited states. Taking Au_60_ as an example, its TA spectra by selective excitation of S_1_ (at 1100 nm, Figure 2a) exhibit a ground-state bleaching (GSB, negative) peak at 1070 nm and a broad excited-state absorption (ESA, positive) beyond 1150 nm. The transient spectra at early delay times consist of a main ESA peak at 1210 nm, which is attributed to the S_1_ population generated immediately upon excitation. The broad ESA (1150–1400 nm, peak at 1270 nm) increases simultaneously with the decay of S_1_. The long-lived lifetime of the final state is ∼320 ns by fitting the ESA from ns-TA, which can be attributed to a triplet state. To better describe the predecessor-successor pair of the ISC process (S_1_ → T_1_), the kinetics at 1210 and 1365 nm were extracted (Figure S4), and they show obvious differences beyond hundreds of ps. Considering the significant overlap of singlet and triplet excited-state spectra, we deduce the triplet state kinetics by scaling the kinetics of 1210 and 1365 nm, which shows a slow rise of ∼1 ns (Figure S4). Thus, we attribute this to the ISC process from S_1_, which is slow and comparable to the fluorescence lifetime (0.90 ns).^41^ The transient spectra of S_1_ and T_1_ states (Figure 2d) are extracted by using a target model, including parallel decay pathways of S_1_: a) directly to the ground state with radiative and nonradiative processes (S_1_ → S_0_), and b) ISC process (S_1_ → T_1_), along with triplet decay (T_1_ → S_0_). Consistent with the analysis of the raw TA map (Figure 2a), along with GSB at 1070 nm, the broad ESA was extracted beyond 1150 nm, with peaks at 1210 nm (assigned to S_1_) and 1270 nm (assigned to T_1_).

**Figure 2:**
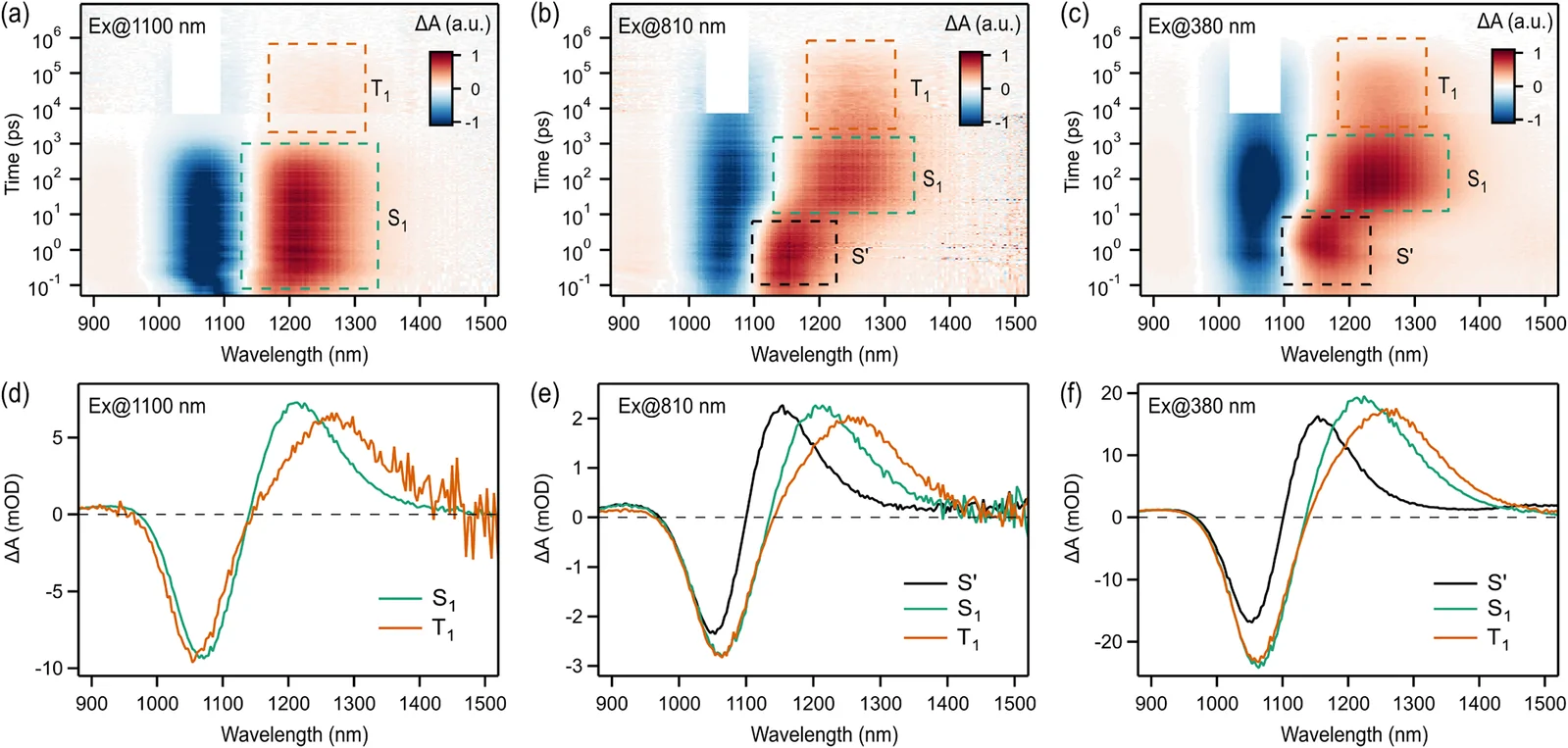
Combined ns and fs transient absorption data map of Au_60_ with selected excitation wavelengths, i.e., 1100 nm for S_1_ excitation (a), 810 nm for the broad range between S_1_ and S_n_ (b), and 380 nm for S_n_ excitation (c). The species-associated spectra of S′, S_1_, and T_1_ (d–f) are extracted by target analysis. Note: the white boxes for ns TA in (a), (b), and (c) are the removal of residual pump-laser peaks of the probe.

The aforementioned PL analysis indicates that the exciton dynamics should differ under S_1_ and S_n_ excitation. Different from S_1_ excitation (Figure 2a), S_n_ excitation (380 nm) gives rise to a new ESA peak at 1150 nm (Figure 2c), which decays in ∼16.9 ps with the rise of S_1_ and T_1_ state populations simultaneously. We attribute this intermediate state to S′, which may contribute to a fast ISC process (high-lying ISC like a “skybridge”) that leads to higher triplet yield and reduced FL due to the dark nature of T_1_. Compared to the S_1_ and S_n_ states observed in the absorption spectra, the S′ state (located between S_1_ and S_n_) possesses a much smaller oscillator strength, leading to weak absorption observed in the steady-state spectra. The kinetics of selected wavelengths (1150, 1250, and 1365 nm) indicate the state evolution (Figure S5). The rise of extracted triplet state kinetics indicates a fast process (∼16.9 ps) followed by a slow process (∼1 ns). Importantly, the exciton dynamics upon S_n_ excitation converge to the same excited-state manifold as that of S_1_ excitation, providing direct evidence that there is no additional emissive species upon S_n_ excitation.

For the 500 to 900 nm weak absorption of Au_60_ (Figure 1a), we selected a series of excitation wavelengths (500, 730, and 810 nm) besides the direct S_1_ excitation (1000 and 1100 nm) and the S_n_ excitation (380 nm) to check the excitation wavelength-dependent properties of Au QRs (Figure 2b and Figures S6–S8). The transient spectra show clearly the S′ peak when excited at high energy (<810 nm), which is ∼0.3 eV higher than the S_1_ state. For long delay times, the excited-state evolution is quite similar to that of direct S_1_ excitation.

For Au_60_, a distinguished GSB signal (∼1070 nm) can be observed in the NIR probe region. Generally, the efficient and unity transition from the singlet to triplet state will not result in the bleaching signal recovering. The kinetics at 1060 nm (blue side of GSB to avoid overlap with ESA) is extracted to indicate the exciton loss during the transition of states (Figure 3). Since the triplet generation occurs in ∼1 ns, we can safely assign that the triplet population contributes to the GSB signal by the tail delay time of fs-TA (∼7 ns). Thus, we can extract the triplet yield by estimating the GSB at 7 ns relative to the initial population of the singlet state. The triplet population related to the ISC process can then be determined. Especially, there is no serious overlap of GSB and ESA for the S_1_ and T_1_ states, since there is no shift of GSB during the normal ISC process (Figure 2). For direct S_1_ excitations (1000 and 1100 nm), the triplet population with normal ISC (S_1_ → T_1_) is ∼ 10%. The dynamics upon excitation at high vibronic states of S_1_ is demonstrated to be the same as that of S_1_ peak excitation (Figure 3 and Figure S9). While for higher energy excitation (excitation wavelength ≤900 nm), the triplet yield is up to ∼60% with both the fast ISC (S′ → T_n_(T_1_)) and normal ISC (S_1_ → T_1_). The triplet yield of S_n_ excitation was calculated by the difference of PL QY, which is consistent with the GSB residual from TA measurements. The triplet lifetime is determined to be ∼320 ns (Figure 3b) with nonradiative processes (weak PH observed for Au_60_). The PH is still weak even though a ∼60% yield of the triplet population results under S_n_ excitation. The intensity of FL is 50× higher than that of PH (i.e., the FL/PH peak area ratio, Figure S2a) when excited by high-energy photons. Thus, we build the dynamics model (see the section on Target Analysis of Au_60_ to Au_114_ QRs) to perform the target global analysis. The same species-associated spectra (SAS, generated by target analysis) of S_1_ and T_1_ were also obtained upon excitation at 810 and 380 nm (Figure 2e, f) with the normal ISC process being ∼920 ps. In addition, the peak of S′ is located at 1150 nm, partially overlapping with the GSB signal.

**Figure 3:**
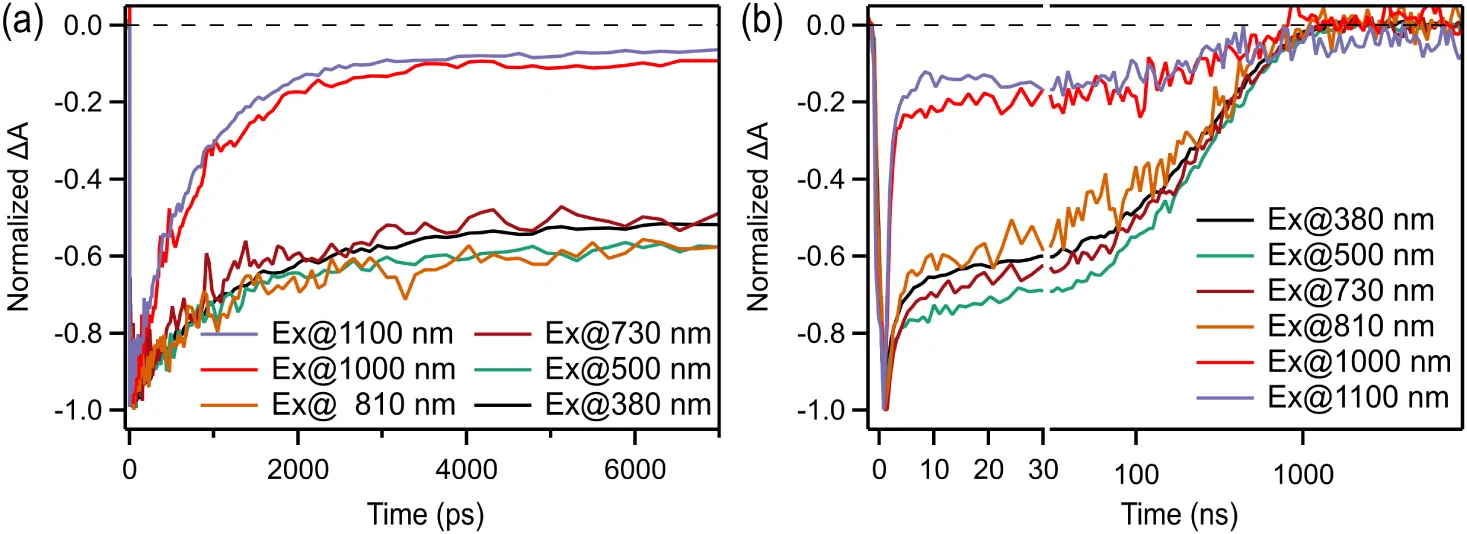
Bleach signals (∼1060 nm) extracted from fs-TA (a) and ns-TA (b) of Au_60_ with different excitation wavelengths.

### Exciton Dynamics of Longer QRs

TA measurements on Au_78_, Au_96_, and Au_114_ were also performed under their corresponding S_1_ and common S_n_ (380 nm) excitation (Figure 4). Upon S_1_ excitation (1300 nm) of Au_78_ (Figure 4a), the GSB and ESA of the S_1_ state decay in 520 ps, with a tiny long-lived triplet population (∼5%), which indicates inefficient ISC from S_1_ to T_1_. However, the S′ state (located at 1450 nm) can be observed when excited at 380 nm (Figure 4b). We can observe the state evolution despite the serious overlap of these two states from ESA peaks close to 1500 nm. The SAS of different excited states are shown in Figure S10. We also performed TA measurements by high vibronic S_1_ excitation (1200 nm) to confirm similar dynamics to that of S_1_ peak excitation (Figure S11). The lifetime of the final state is up to 83 ns with higher yield when excited at 380 nm, which is attributed to the triplet state (Figure S12). The decay kinetics of GSB (Figure S13) show a fast decay in several ns with exciton loss of more than 80% upon S_1_ excitation, which is induced by (S_1_ → S_0_) via radiative and nonradiative processes. We did not observe fast decay upon S_n_ excitation because of the fast ISC process, which is limited by the instrument response function (IRF, ∼1 ns of our ns-TA setup). Limited by the probe light range (up to ∼1600 nm only), we cannot observe the distinguishing ESA signatures of S′, S_1_, and T_1_ on the red side of the bleaching peak (>1600 nm) for Au_96_ and Au_114_ (Figure 4c–f and Figure S14). However, the exciton lifetime under S_n_ excitation is much longer than that of S_1_ excitation, indicating triplet formation via S′. The triplet lifetime was determined to be 21 ns for Au_96_ and 13 ns for Au_114_, which are consistent with previous TA results probed in the visible region.^40^ In contrast, the exciton lifetime under S_1_ excitation is only 76.1 ps for Au_96_ and 61.2 ps for Au_114_, which indicates fast nonradiative decay to the ground state (S_1_ → S_0_) without populating the triplet state. From the GSB kinetics, one sees a faster decay and higher exciton loss with increasing length of QRs upon S_1_ excitation (Figure 4g). However, a much higher yield and shorter lifetime of the triplet state are observed when excited at 380 nm (Figure 4h and Figure S15).

**Figure 4:**
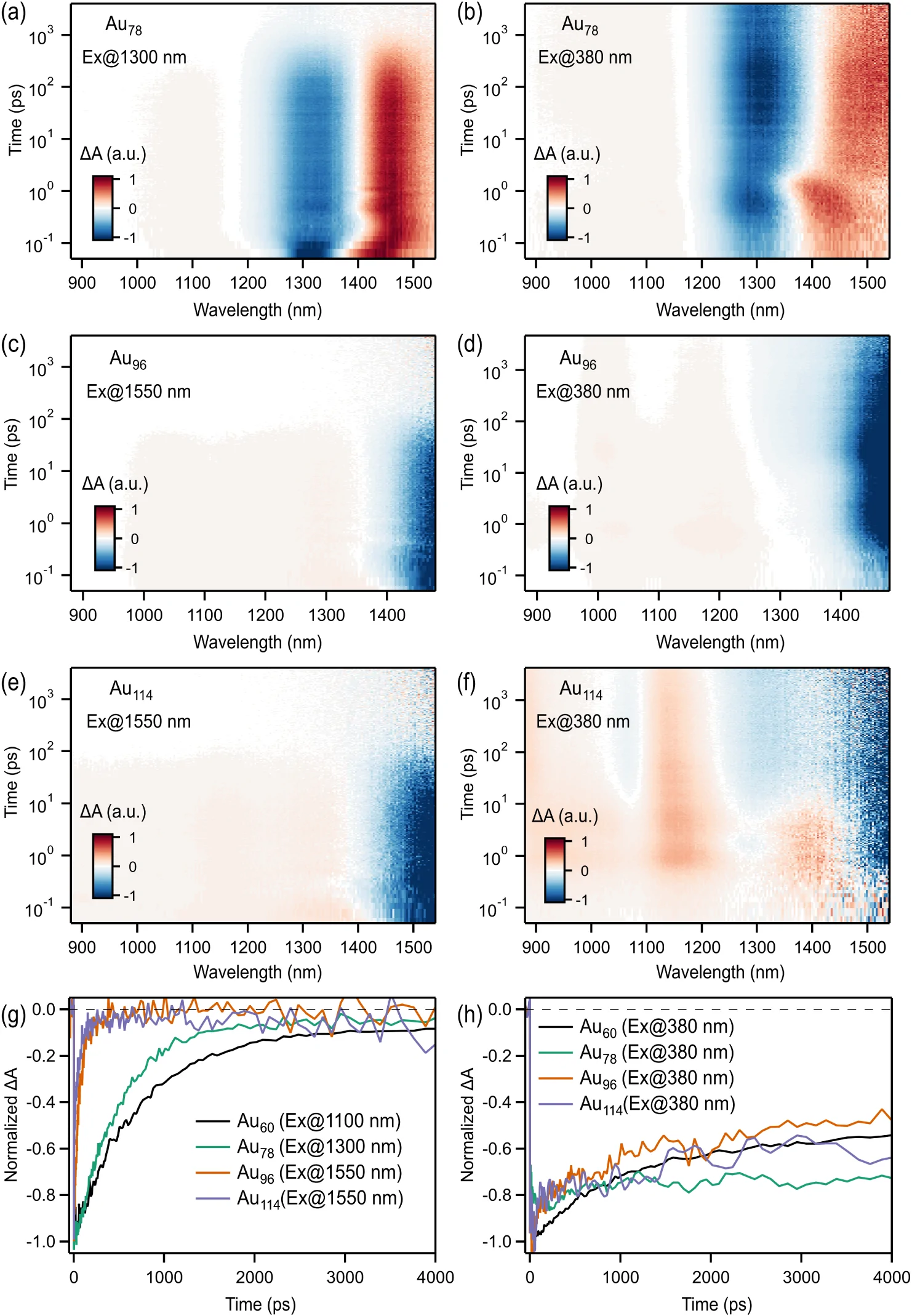
Transient absorption spectra of Au_78_ (a, b), Au_96_ (c, d), and Au_114_ (e, f) with S_n_ excitation (380 nm) and S_1_ excitation (1300 nm for Au_78_, 1550 nm for Au_96_ and Au_114_; note 1550 nm is the maximum available wavelength in our setup). The bleach signal decay (g, h) of these four QRs indicates more triplet populations under the S_n_ excitation.

### Target Analysis of Au_60_ to Au_114_ QRs

The lifetimes of excited states can be extracted by target analysis based on the complicated model comprising the “skybridge” ISC from the intermediate state (S′) to a high triplet state and the normal ISC from S_1_ to T_1_ (Scheme 1). By combining the results of PL and TA measurements, we put forth a full Jablonski diagram to describe the dynamics of QRs, in particular, the decay of S′ includes the fast IC process (S′ → S_1_) and the fast ISC process (S′ → T_*n*≥2_ → T_1_). Given the fast process, we ignore the nonradiative process directly to the ground state; thus, we have the following equation,

$$k_{S^{\prime }}=k_{S^{\prime },IC}+k_{S^{\prime },ISC}$$

**Scheme 1:**
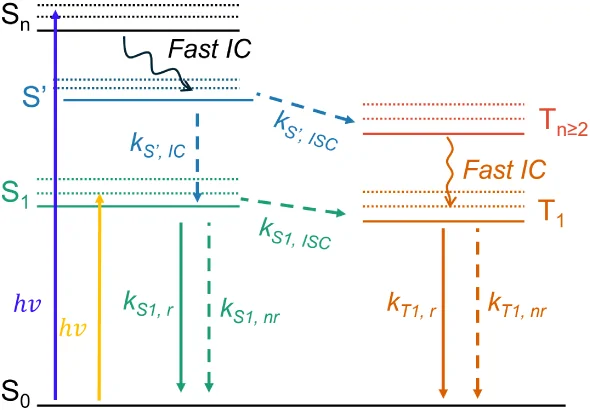
Kinetic Model for the Au QRs of Au_60_ to Au_114_

Different from the decay of S′ state, we should include the nonradiative processes for the S_1_ or T_1_ state, considering the exciton loss during the state transition process. Thus, the kinetics equations for S_1_ or T_1_ are as follows:

$$k_{S_{1}}=k_{S_{1},r}+k_{S_{1},ISC}+k_{S_{1},nr}$$

$$k_{T_{1}}=k_{T_{1},r}+k_{T_{1},nr}$$

Since no obvious phosphorescence is observed for the long QRs, we attribute that the dark triplet formation is the main reason for QY loss when high-lying S_n_ is excited. Meanwhile, the bleaching signals from TA indicate that the exciton population decays during the excited-state transition. Taking Au_60_ as an example, the QY can be up to 20.9% based on 100% population of S_1_ under direct S_1_ excitation. As shown in Figure 4g, the triplet population is only ∼10% (estimated from the bleach signal residual up to 5 ns) under S_1_ excitation, indicating that the normal ISC process (S_1_ → T_1_) is ∼ 10% of the S_1_ decay. Thus, the nonradiative process is ∼69.1%. Considering S_n_ excitation, the QY decreases to 9.5%. Thus, we assume that 45% (i.e., the QY ratio of 9.5/20.9) of the indirect population of S_1_ is from S′ decay via an IC process (∼16.9 ps). In other words, 55% of the population of T_1_ can be obtained via the skybridge ISC process (S′ → T_n≥2_ → T_1_). Based on this mechanism, we can calculate the triplet population to be ∼60%, that is, 0.55 + 0.45 × 0.10 = 0.60, which is consistent with the GSB signal under S_n_ excitation (Figure 4h). On a note, we have to sum the nonradiative and radiative processes together for the target analysis. The target fitting parameters for different decay pathways are summarized in Table S1 for Au_60_. The corresponding populations of excited states are shown in Figure S16, indicating the state evolution with exciton loss; the rate constants are compiled in Table 1.

**Table 1:** Summary of the Rate Constants (*k*_IC_, *k*_ISC_, *k*_r_, and *k*_nr_) of the QRs

	High-lying intermediate, S′		Lowest singlet, S_1_			Lowest triplet, T_1_	
	$k_{S^{\prime },IC}$ (×10^10^ s^–1^)	$k_{S^{\prime },\mathrm{ISC}}$ (×10^10^ s^–1^)	$k_{S_{1},r}$ (×10^8^ s^–1^)	$k_{S_{1},ISC}$ (×10^8^ s^–1^)	$k_{S_{1},nr}$ (×10^8^ s^–1^)	$k_{T_{1},r}$ (×10^4^ s^–1^)	$k_{T_{1},nr}$ (×10^6^ s^–1^)
Au_60_	2.7	3.3	2.3	1.1	7.5	∼0	3.1
Au_78_	1.7	11.4	3.1	0.96	15.2	∼0	12
Au_96_	1.1	4.0	2.5	∼0	128	∼0	48
Au_114_	0.5	4.1	1.6	∼0	162	∼0	77

We performed the same analysis for Au_78_, Au_96_, and Au_114_. The corresponding rates and yields are determined (Tables 1 and 2, and Table S2). For the QRs with large AR, the radiative process of T_1_ triplet state is ignored since no PH was observed, except for the weak PH from Au_60_.

**Table 2:** Relative Ratio of Decay Pathways of Each Excited State for Au_60_ to Au_114_ QRs

	S′		S_1_			T_1_			
	$\Phi_{S^{\prime },IC}$	$\Phi_{S^{\prime },ISC}$	$\Phi_{S_{1},r}$	$\Phi_{S_{1},\mathrm{ISC}}$	$\Phi_{S_{1},nr}$	$\Phi_{T_{1},r}$	$\Phi_{T_{1},nr}$	Triplet yield at S_1_ excitation	Triplet yield at S_n_ excitation^a^
Au_60_	0.45	0.55	0.21	0.10	0.69	0	1	0.10	0.60
Au_78_	0.13	0.87	0.16	0.05	0.79	0	1	0.05	0.88
Au_96_	0.21	0.79	0.02	0	0.98	0	1	0	0.79
Au_114_	0.11	0.89	0.01	0	0.99	0	1	0	0.89

a Under S_n_ excitation, triplet yield $\Phi_{T}=\Phi_{S^{\prime },ISC}+\Phi_{S^{\prime },IC}\times \Phi_{S_{1},\mathrm{ISC}}$ .

From the experimental FL and PH peaks, the energy gap between S_1_ and T_1_ (Δ*E_ST_*) is ∼ 0.22 eV for both Au_42_ ^10^ and Au_60_ (see Figure S2a, estimated by peak to peak separation). In theoretical work,^43^ the Δ*E_ST_* gap was calculated to be 0.39 eV for Au_42_ and 0.57 eV for Au_60_, and the ISC rates were calculated to be 1.23 × 10^8^ s^–1^ and 8.68 × 10^8^ s^–1^, respectively. The theoretical simulations ignored the nonradiative process from S_1_,^43^ but our experimental work includes the nonradiative process as a process of exciton loss, which competes with the radiative and ISC processes of S_1_. For the long QRs (Au_60_, Au_78_, Au_96_, Au_114_), even though $k_{S_{1},r}$ is ∼10^8^ s^–1^ (Table 1), the increasing $k_{S_{1},\mathrm{nr}}$ results in fast decay to the ground state; thus, the normal ISC process (S_1_ to T_1_) is significantly suppressed by the nonradiative process (up to tens of ps) with increasing length of QRs.

Based on the PL and TA measurements, we have identified two separate ISC processes upon S_n_ excitation for the QRs, in which the fast ISC from the S′ state is high-lying (“skybridge”), which is 2 orders of magnitude faster than the normal ISC (S_1_ to T_1_) process. Therefore, under S_n_ excitation, QRs show much more complicated exciton dynamics than previous APNCs.^14,16^ Comparable to the fast IC process within the singlet manifold (of order 10^10^ s^–1^), the skybridge ISC (of order 10^10^ s^–1^) from the S′ state contributes to most of the triplet population upon S_n_ excitation. For Au_60_, the total triplet yield is ∼60%, in which the triplet yield of up to 55% (absolute) is populated via the fast ISC. For the longer QRs, the fast ISC process contributes to ∼90% for Au_60_, ∼99% for Au_78_, and 100% for Au_96_ and Au_114_, whereas the normal ISC is essentially suppressed by faster *k*_nr_ of the S_1_ state. While one would expect to see stronger triplet emission due to the higher yield via the fast ISC, the relatively large nonradiative process from the T_1_ state overwhelms the PH emission even with a higher triplet population; thus, no PH is observed.

The above results of excitation wavelength-dependent exciton dynamics and QY for the QRs clearly show anti-Kasha–Vavilov behavior,^47–49^ that is, unlike the typical process of S_n_ → S_1_ → T_1_ (the Kasha–Vavilov behavior), a branching process (S_n_ → S′ → T_*n*≥2_ → T_1_) is discovered in the QRs, which may follow the El-Sayed rule with higher spin–orbit coupling (SOC) between the S′ state and the triplet manifold,^50,51^ and another factor is the possibility of a smaller gap between S′ and T*_n_*_≥2_ than that between S_1_ and T_1_ (∼0.2 eV gap). These two factors can accelerate the high-lying ISC. Although the emission still comes from the lowest excited states, the identified metastable excited state (S′) holds potential for future exploration of new photochemistry that requires more energy input and higher electrochemical potential.^21,30,31^ In addition, the effective modulation of the triplet T_1_ population will benefit photoexcited electron and energy transfer,^3,4,52,53^ as well as other optoelectronic and photonic applications.

Finally, it is worth noting that the S_1_ exciton is strongly anisotropic, in that the transition dipole’s component along the longitudinal direction (*d*_*z*_) is significantly larger than the *d*_*x*_ and *d*_*y*_ components.^40,45^ In contrast, the S_n_ exciton is isotropic,^45^ and given its nature of being nearly constant (at ∼400 nm regardless of the QR geometry), this exciton is tentatively assigned to a transition from gold *d*-states (forming a quasi-*d*-band with many gold atoms in the QR) to sulfur *p*-states, because these two groups of states are relatively stationary and do not shift with the QR size/geometry. The excitation wavelength-dependent TA measurements revealed that excitation at S_1_ or S_n_ converges to the same excited state manifolds after 10s of ps. No additional excited states beyond S′ were observed at S_n_ excitation. After the initial fast relaxation (several 10s of ps), the dynamics of both excitation scenarios exhibit identical transient spectral signatures (consisting of S_1_ and T_1_ states only), see Figure S17 for TA spectral comparison of S_1_ and T_1_. Furthermore, the fluorescence peak wavelength and width are identical under S_1_ and S_n_ excitation, indicating the same emissive S_1_ state (Figure S18). In the case of Au_60_, the weak phosphorescence observed upon S_n_ excitation is attributed to the S′-mediated fast ISC process, which largely increases the triplet yield but does not introduce a different emissive state. Both PL and TA spectral comparisons upon respective S_1_ and S_n_ excitation provide evidence for the same emissive state and confirm efficient coupling between the two excitonic modes.

## Conclusion

In summary, the excitation wavelength-dependent photoluminescence and exciton dynamics of the periodic QRs of Au_60_, Au_78_, Au_96_, and Au_114_ are investigated. Unlike other APNCs, the exciton dynamics of the QRs can be modulated by the excitation wavelength, in which S_1_ excitation gives rise to a much higher QY (about 2 to 10 times) than that of S_n_ (400 nm) excitation for the investigated QRs. On the other hand, much higher triplet yields result upon S_n_ excitation, which holds potential for T_1_ utilization such as energy and electron transfer applications, as well as potential hot-state utilization in new photochemistry. With respect to emission, the QRs show fluorescence only, even with higher triplet populations at S_n_ excitation. Mechanistically, a metastable intermediate energy state (S′) with signature peaks in transient absorption is observed. This S′ state leads to anti-Kasha–Vavilov behavior, that is, a branching of the S_n_ to S_1_ process (the regular relaxation) with unusual relaxation of S_n_ to S′ to T_*n*≥2_, which is first observed in APNCs due to the anisotropic shape of QRs. Our study reveals a design principle (i.e., by the rod length or aspect ratio) for QRs with anti-Kasha–Vavilov properties. Although the photoluminescence is still from the lowest energy states, the metastable intermediate S′ state can be harnessed for potential applications in energy/electron transfer, electrochemistry, and photocatalysis. Overall, such QRs provide an efficient way to manipulate the triplet population, which may contribute to NIR-II photon upconversion, NIR-II photocatalysis, and biorelated applications.

## Supplementary Material



## Notes

Experimental section, including the chemicals, synthesis of QRs, steady-state and time-resolved PL spectroscopy, fs and ns transient absorption, target analysis, supporting figures S1–S18, and tables S1–S2 (PDF)
