# Supplementary material for: Gold Quantum Rods: Modulation of Singlet and Triplet Exciton Populations by the Rod Length
Source: J Am Chem Soc. 2026 Aug 12;148(33):36062–70. doi: 10.1021/jacs.6c10470 (PMC13523701; doi:10.1021/jacs.6c10470)
Supplement: Supplementary file 1 [file ja6c10470_si_001.pdf]

(Supporting Information)

Gold Quantum Rods: Modulation of Singlet and Triplet Exciton Populations by the Rod Length

Guiying He,[#,1] Lianshun Luo,[#,1] Avirup Sardar,[1] Weijie Ji,[1] Mircea Cotlet,[2] and Rongchao Jin*,[1]

[1]Department of Chemistry, Carnegie Mellon University, Pittsburgh, Pennsylvania 15213, United States
[2]Center for Functional Nanomaterials, Brookhaven National Laboratory, Upton, New York 11973, United States

[#]Equal contribution; *Corresponding author, E-mail: rongchao@andrew.cmu.edu

Experimental Section

Chemicals. Tetrachloroauric (III) acid ($HAuCl_4 \cdot 3H_2O$, 99.999% metal basis, Aldrich), phenylethyl mercaptan (PET, >99%, Aldrich), sodium borohydride (powder, ≥98%, Sigma), benzimidazole (98%, Aldrich), 2-bromopropane (99%, Aldrich), dimethyl sulfide (SMe_2, >99%, Aldrich), potassium carbonate (K_2CO_3, 99%, Aldrich), toluene-d8 (99 atom % D, Sigma), tetrachloroethylene (C_2Cl_4), methanol, ethanol, ethyl ether, ethyl acetate, pentane, dichloromethane (DCM), chloroform, *n*-hexane and acetonitrile (HPLC grade for all solvents) were used as received. Thin-layer chromatography (TLC) plates were from iChromatography (silica gel, 250 μm).

Synthesis of fluorescent quantum rods

The details of synthesis and characterization of nanorods clusters were reported previously.[1] Briefly, $^{i}Pr_2$-bimy·AuBr (120 mg, 0.25 mmol) and PET thiol ($HSCH_2CH_2Ph$, 67 μL, 0.5 mmol) were dissolved in mixed chloroform and ethanol (15 + 5 mL). The mixture gradually turned cloudy white (Au^I-PET formed). After 1 hour of stirring, the suspension was reduced to nanoclusters (NCs) by the addition of sodium borohydride (95 mg, 2.5 mmol, dissolved in 5 mL ice-cold H_2O) under darkness. A black solution was formed, and the reaction was allowed to proceed for 6 hours, then the solvent was removed under reduced pressure. The mixture of Au NCs was thoroughly washed with methanol, extracted with DCM, and concentrated for TLC separation. For the latter, the mixture of Au NCs was first pipetted onto a TLC plate, and the separation was conducted in a developing tank (developing solvent 1:1 (v/v) DCM:*n*-hexane). Four bands corresponding to $Au_{60}(PET)_{44}$, $Au_{78}(PET)_{56}$, $Au_{96}(PET)_{68}$, and $Au_{114}(PET)_{80}$ were cut off and dissolved in DCM for characterization. Plate-shaped crystals of $Au_{60}(PET)_{44}$ were obtained via evaporation of acetonitrile into a toluene solution of the nanoclusters at room temperature for two weeks.

Optical measurements

UV-vis-NIR absorption spectra of nanoclusters were collected with a UV-3600 Plus UV-vis-NIR spectrophotometer (Shimadzu, detection region: 185-3300 nm).

Steady-state photoluminescence (PL) spectra were recorded using a FLS-1000 spectrofluorometer (Edinburgh Instruments). Both excitation and emission slit bandwidths were set to 10 nm. A photomultiplier tube PMT-1700 detector (cooled to -80 °C by liquid nitrogen) was used for Au_{60} and Au_{78} QRs. The PL peaks of Au_{96} and Au_{114} were beyond the PMT-1700 range, so they were measured by an InAs detector with lock-in amplification (detector range: up to 3000 nm).

The PL lifetime was measured by time-correlated single photon counting (TCSPC) with PMT-1700 detector (limited to Au_{60}, Au_{78}) on the same instrument.

The absolute quantum yield (up to 1650 nm with PMT-1700 detector) were measured using an integrating sphere. First, a blank spectrum was recorded by placing a cuvette containing only the solvent in the integrating sphere with five repeated emission scans, starting from 20 nm longer than the excitation wavelength to the longest wavelength of the detector. Next, the blank was replaced with a dilute solution of Au QRs adjusted to an OD of 0.1 at the excitation wavelength. The emission scan was then repeated five times over the same spectral range. PLQY was determined using the software of FLS-1000. The absolute quantum yields of Au_{60} and Au_{78} in toluene-d8 under S_1 excitation were determined. The relative quantum yields of other nanoclusters in different excitation wavelengths beyond the coverage of PMT-1700 detector were measured by using a reference standard under the same instrumental setting.

Transient absorption (TA) measurements were performed using a broadband pump–probe setup powered by a 1 kHz Ti:Sapphire laser system (Spitfire, Spectra-Physics). Pump wavelengths were generated by a commercial optical parametric amplifier (TOPAS-C, Light Conversion). For femtosecond TA, the NIR supercontinuum probe was produced by focusing the 800 nm fundamental pulse into a sapphire plate and then split into signal and reference beams. The time resolution of fs TA is ~200 fs. The pump–probe delay was controlled via a mechanical delay line. For nanosecond TA, the probe was generated using a fiber laser (Leukos), with delay times up to microseconds controlled by an electronic delay configuration. The TA signals between 1020 nm and 1090 nm are removed because of the residual pump peak of the probe laser. All fs- and ns-TA experiments were conducted in toluene with the optical density adjusted to ~0.2 OD (2 mm cuvette) at the S_1 excitation wavelength. The polarization of pump and probe pulse was set to magic angle (54.7°) to measure isotropic signals. The transient absorption spectra were analyzed using the publicly available program Glotaran based on the statistical fitting package TIMP.[2,3] A branched target model (Scheme 1 in main text) was performed to extract the exciton lifetimes.

Supporting Figures and Tables:

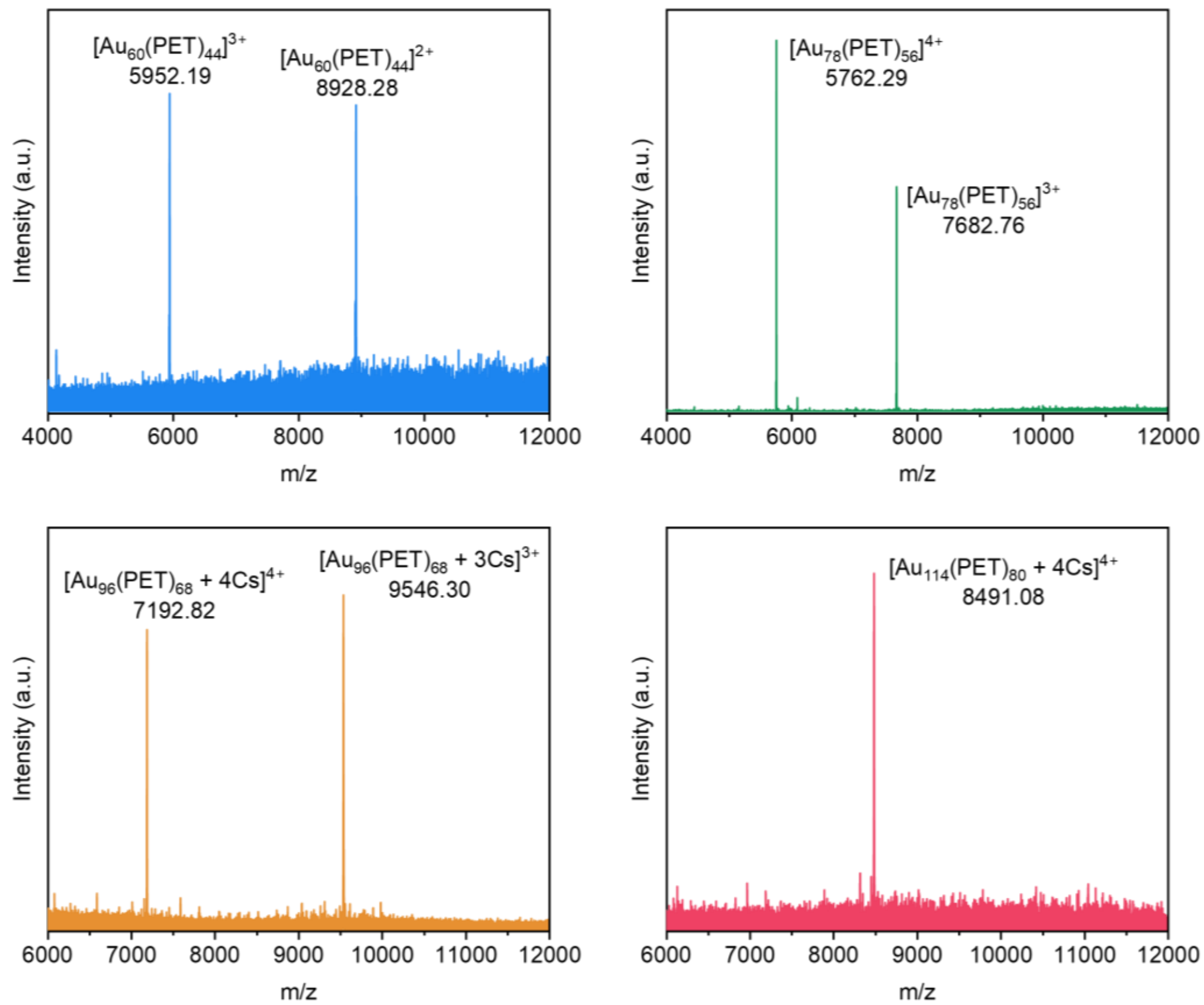

Figure S1. Electrospray ionization mass spectrometry (ESI-MS) of Au_{60}, Au_{78}, Au_{96} and Au_{114}. Both Au_{60} and Au_{78} were analyzed in CH_2Cl_2 without the addition of cesium acetate (CsOAc), while Au_{96} and Au_{114} were analyzed by adding CsOAc for better signals (CH_2Cl_2 : 50 mM CsOAc in MeOH 20 : 1).

The mass peaks are compared with formula-based calculations below.

$Au_{60}(SCH_2CH_2Ph)_{44}$:

peak at 5952.19 = $[Au_{60}(SC_8H_9)_{44}]^{3+}$ (cal: 5951.94)

peak at 8928.28 = $[Au_{60}(SC_8H_9)_{44}]^{2+}$ (cal: 8927.91)

$Au_{78}(SCH_2CH_2Ph)_{56}$:

peak at 5762.29 = $[Au_{78}(SC_8H_9)_{56}]^{4+}$ (cal: 5761.98)

peak at 7682.76 = $[Au_{78}(SC_8H_9)_{56}]^{3+}$ (cal: 7682.64)

$Au_{96}(SCH_2CH_2Ph)_{68}$:

peak at 7192.82 = $[Au_{96}(SC_8H_9)_{68}\mathbf{Cs_4}]^{4+}$ (cal: 7192.91)

peak at 9546.30 = $[Au_{96}(SC_8H_9)_{68}\mathbf{Cs_3}]^{3+}$ (cal: 9546.24)

$Au_{114}(SCH_2CH_2Ph)_{80}$:

peak at 8491.08 = $[Au_{114}(SC_8H_9)_{80}\mathbf{Cs_4}]^{4+}$ (cal: 8490.93)

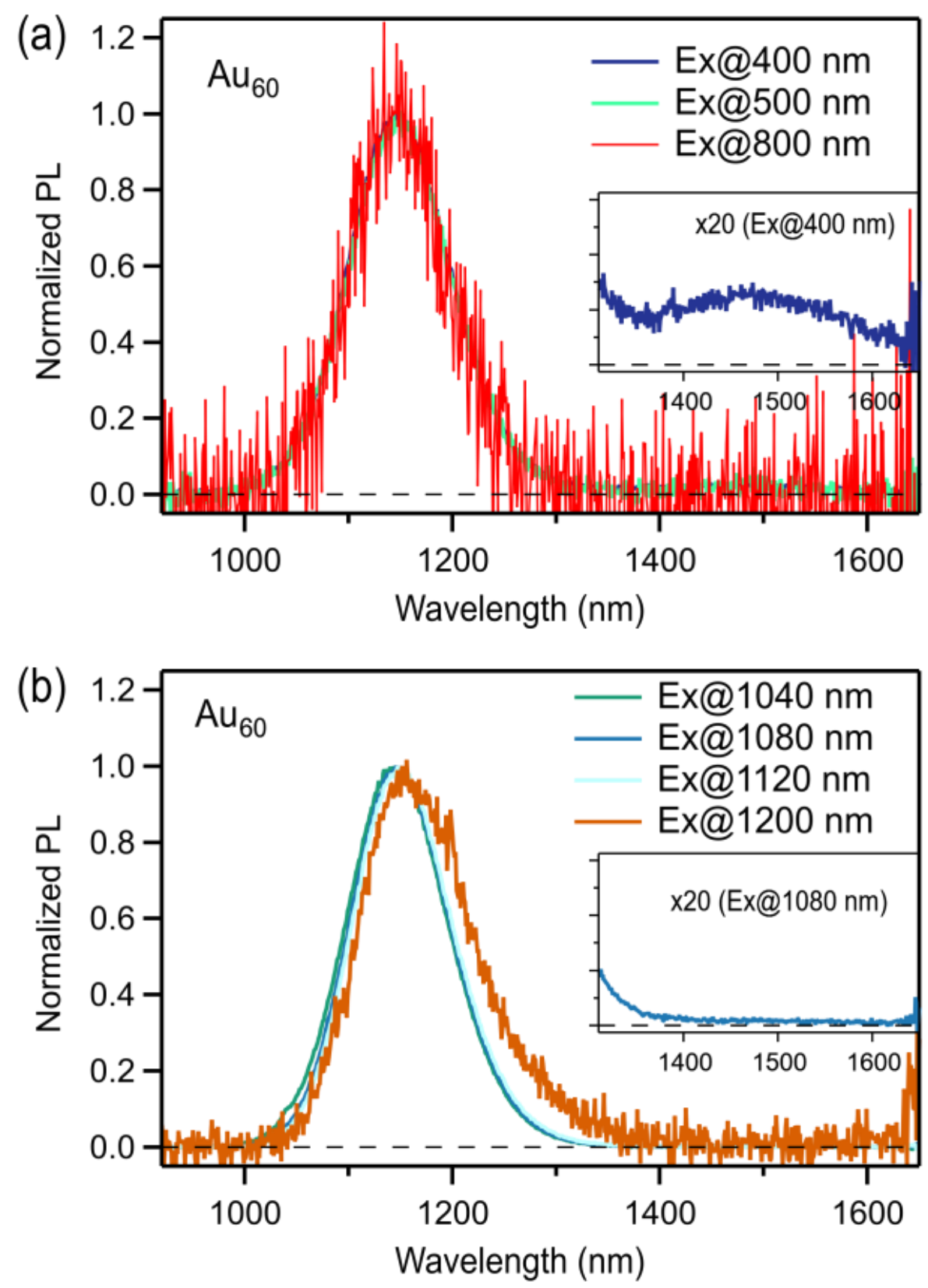

Figure S2. Normalized photoluminescence spectra of Au_{60} with different excitation wavelengths, (a) excitations at higher energy than S_1, and (b) excitations around S_1.

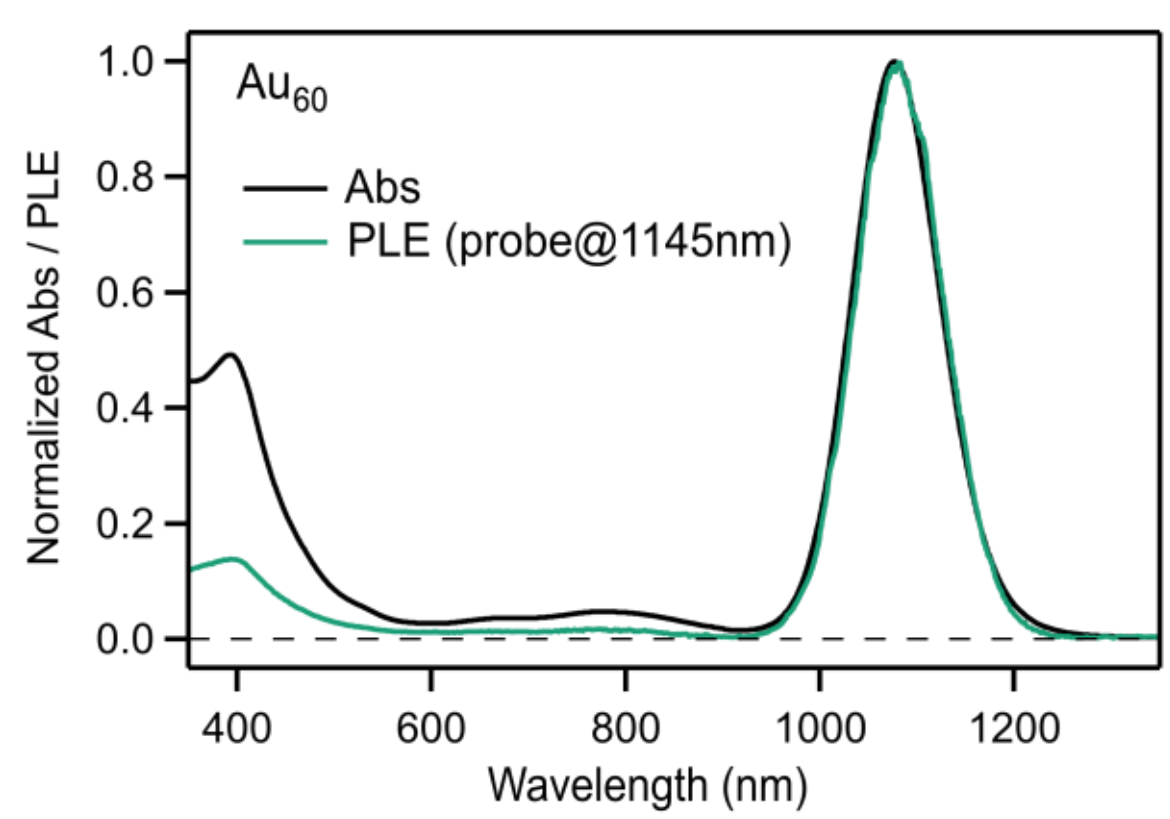

Figure S3. Normalized absorption and PL excitation spectra of Au_{60} (the PLE spectrum corresponds to emission peak of 1145 nm).

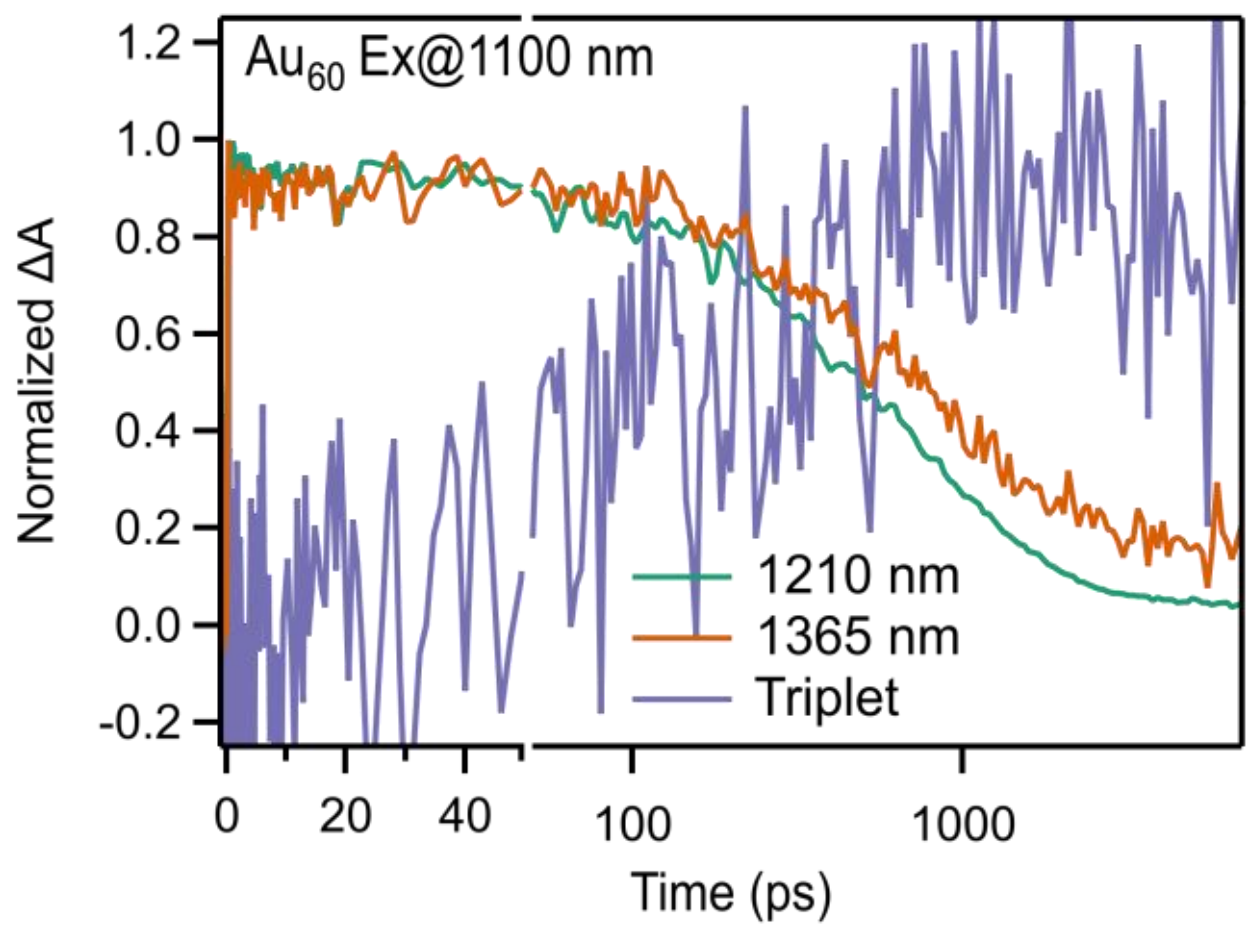

Figure S4. The kinetic cuts at selected wavelengths of Au_{60} excited at 1100 nm. Due to serious overlap of singlet and triplet states, we obtained triplet kinetics by subtracting the scaling kinetics of 1210 nm from the kinetics of 1365 nm, which shows a slow rise ~1 ns (violet curve).

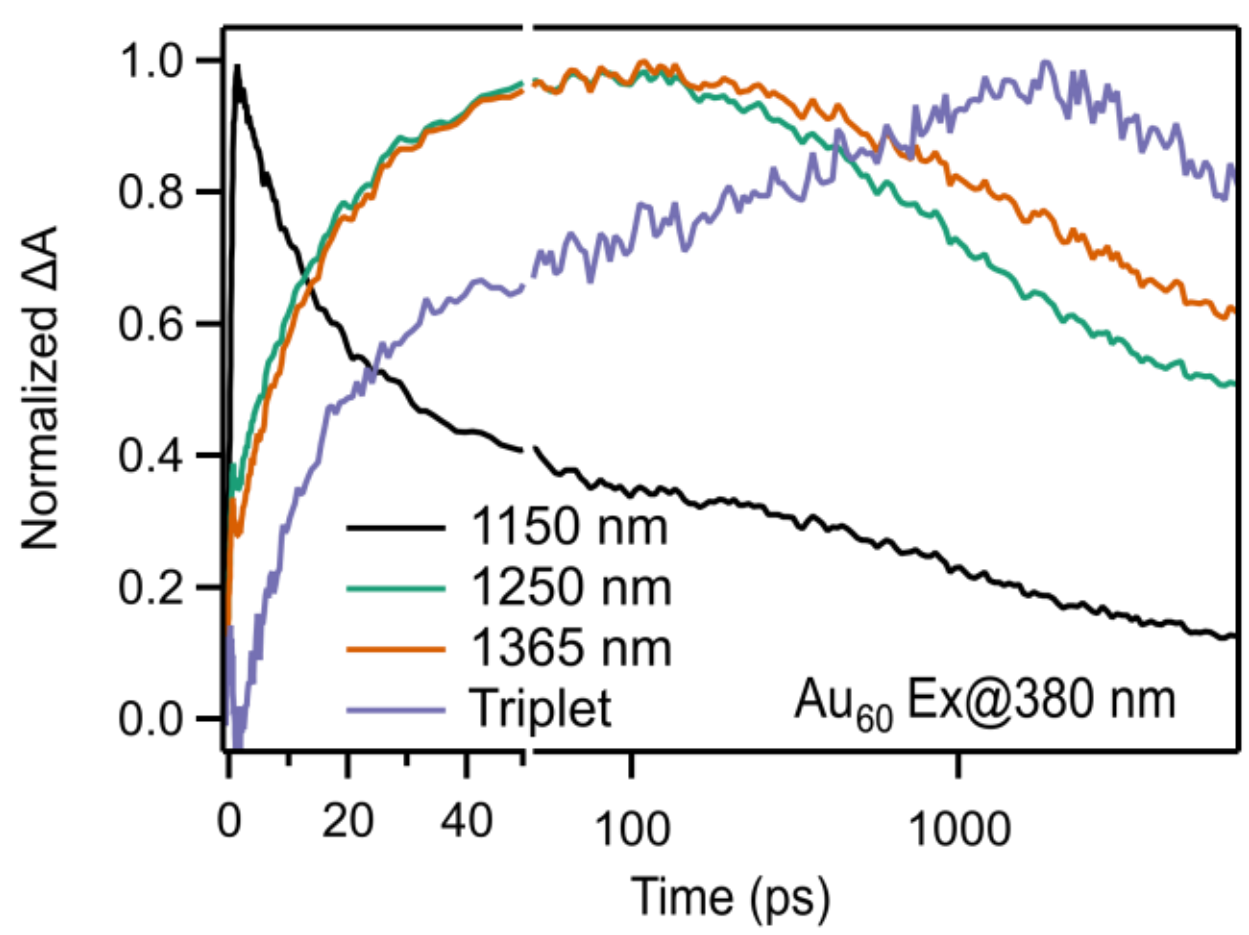

Figure S5. The kinetic cuts at selected wavelengths of Au_{60} excited at 380 nm. Due to serious overlap of singlet and triplet states, we obtained triplet kinetics by subtracting the scaling kinetics of 1250 nm from the kinetics of 1365 nm, which shows a fast (~17 ps) and a slow (~1 ns) rise.

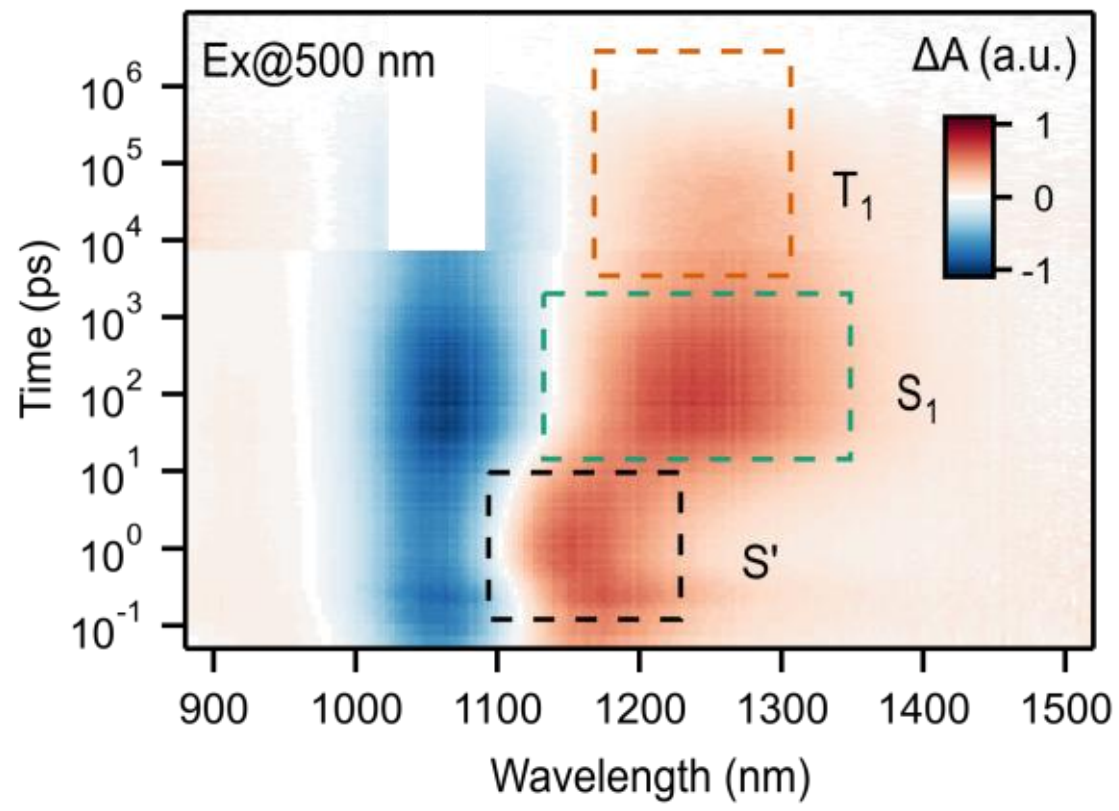

Figure S6. Transient absorption of Au_{60} excited at 500 nm (the fs-TA and ns-TA are joined). Note: in ns-TA, the residual pump-laser peak (around 1050 nm) for the ns probe is removed, hence, displaying a blank region; same below.

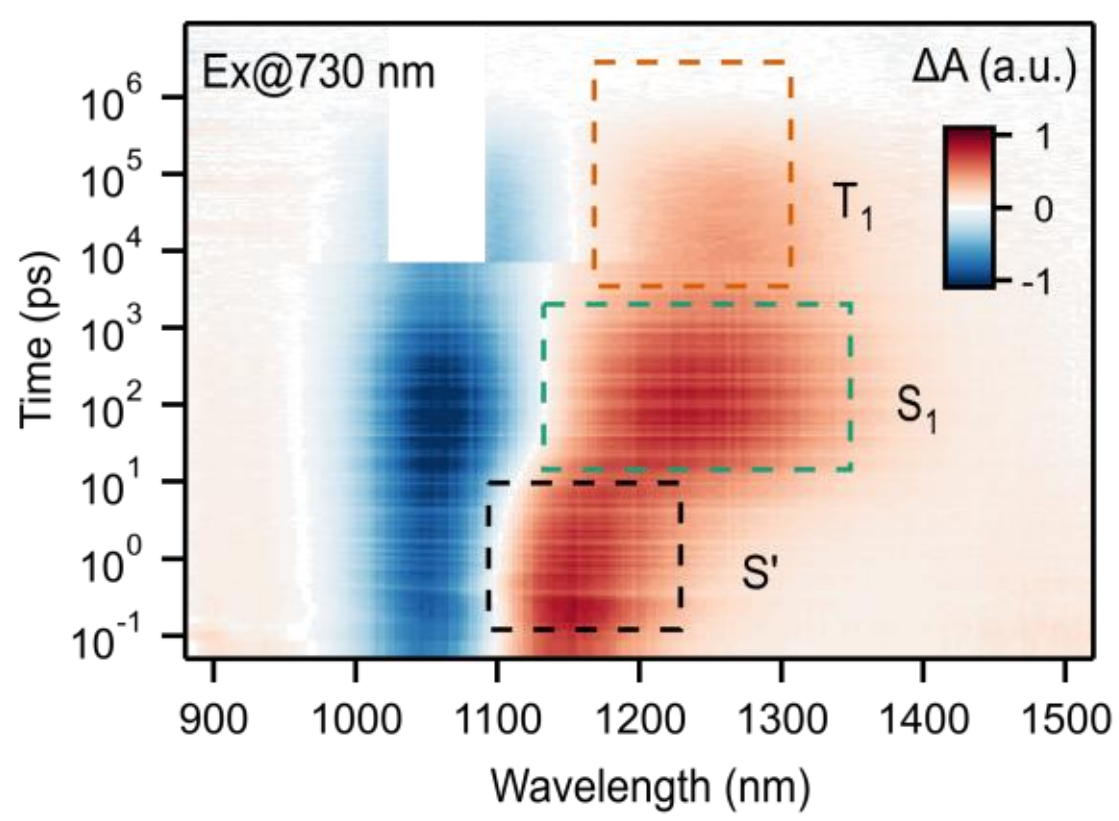

Figure S7. Transient absorption of Au_{60} excited at 730 nm (the fs-TA and ns-TA are joined).

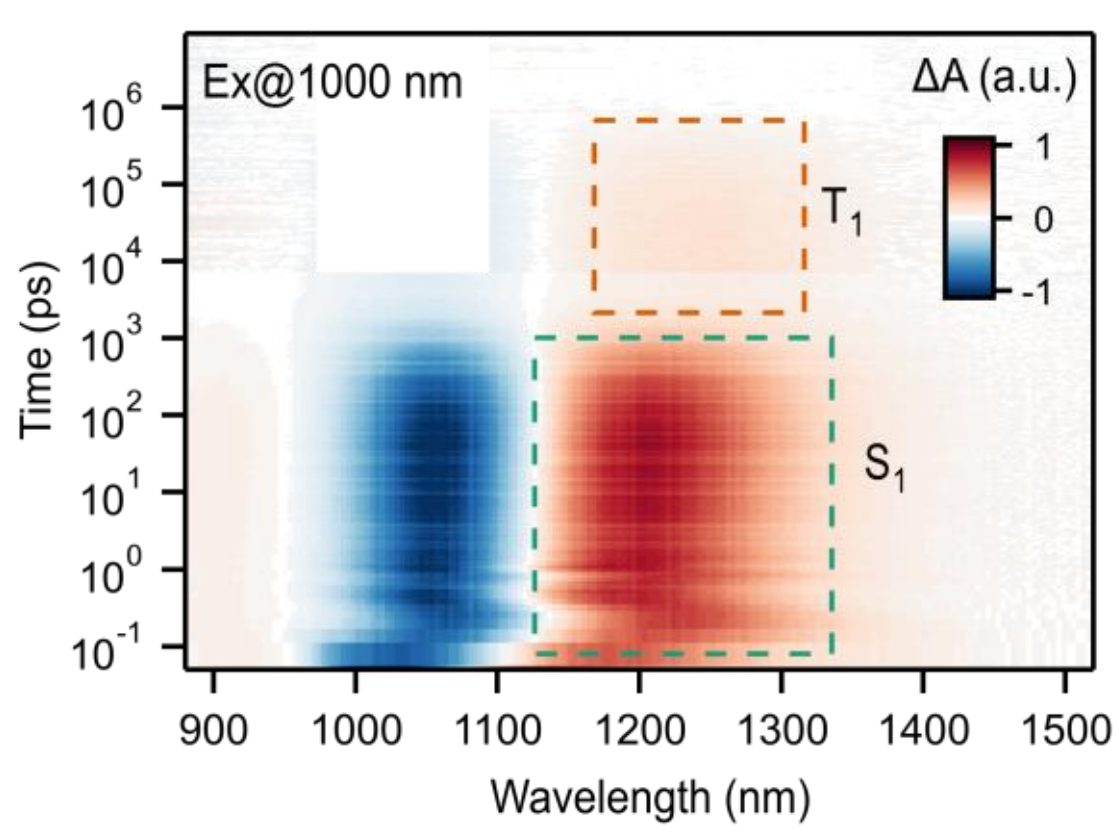

Figure S8. Transient absorption of Au_{60} excited at 1000 nm (the fs-TA and ns-TA are joined).

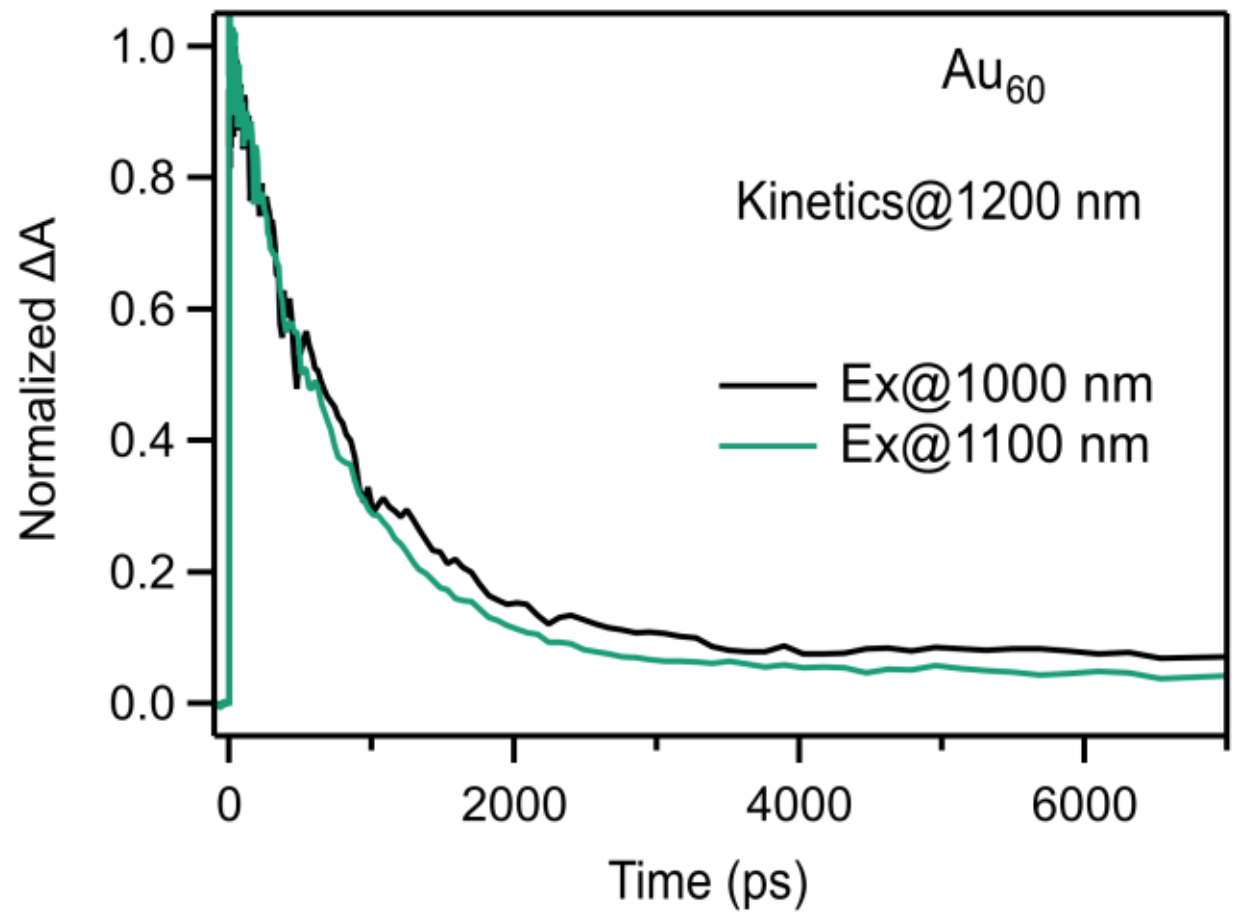

Figure S9. The kinetic cuts of 1200 nm for Au_{60} under excitation of S_1 at 1000 nm (90 meV or 725 cm^{-1} higher than the 1078 nm absorption maximum) and 1100 nm (23 meV lower), respectively.

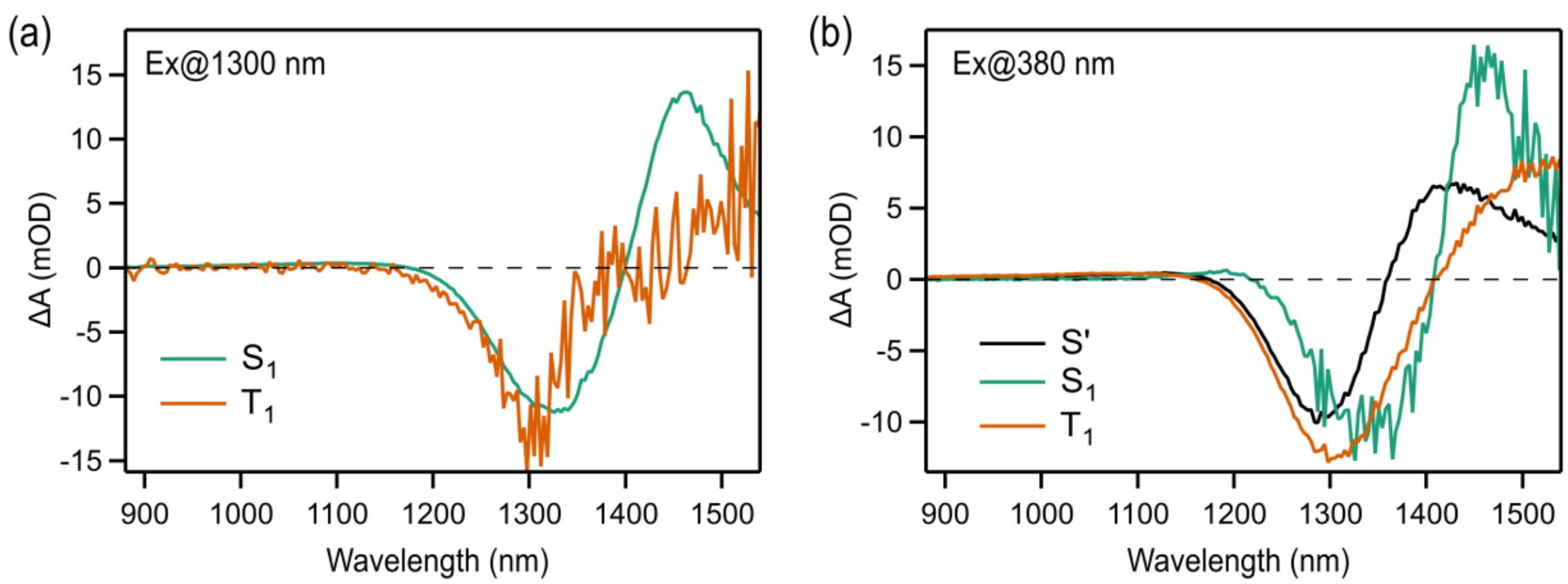

Figure S10. The species associated spectra of Au_{78} excited at 1300 nm (a), and 380 nm (b), extracted by target analysis.

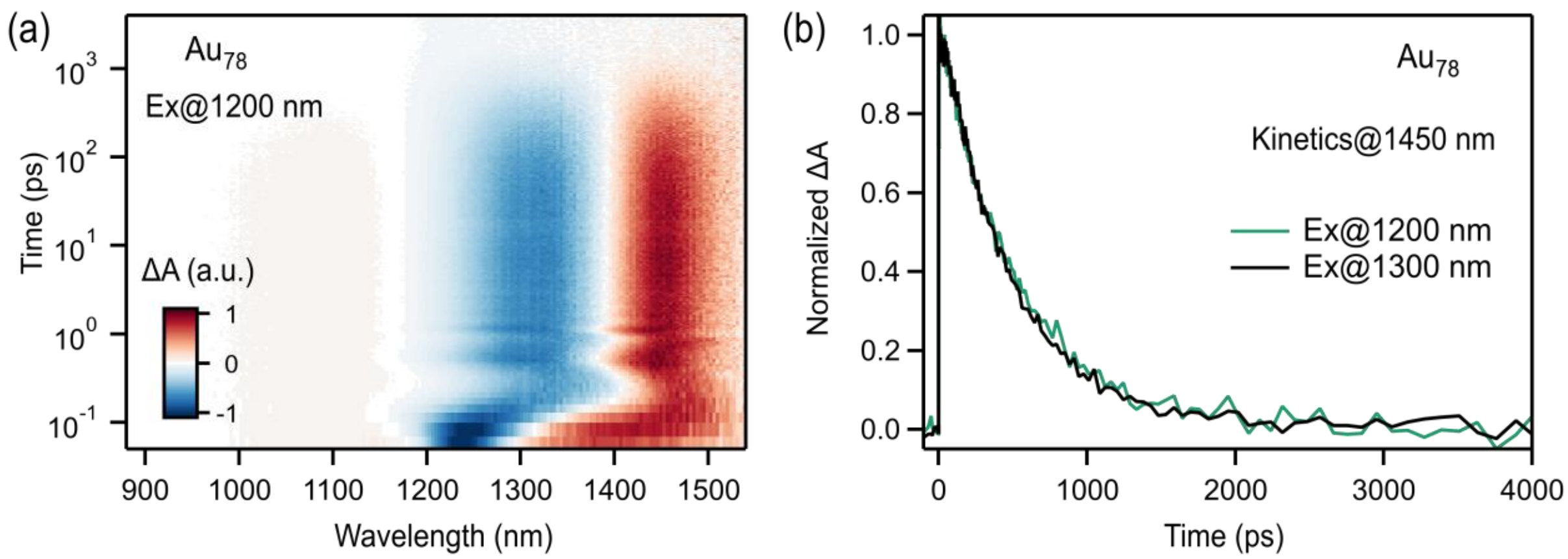

Figure S11. (a) fs-TA of Au_{78} excited at 1200 nm (94 meV or 758 cm^{-1} higher than the 1320 nm absorption maximum), and (b) the kinetic cuts of 1450 nm under excitation of S_1 at 1200 nm and 1300 nm, respectively.

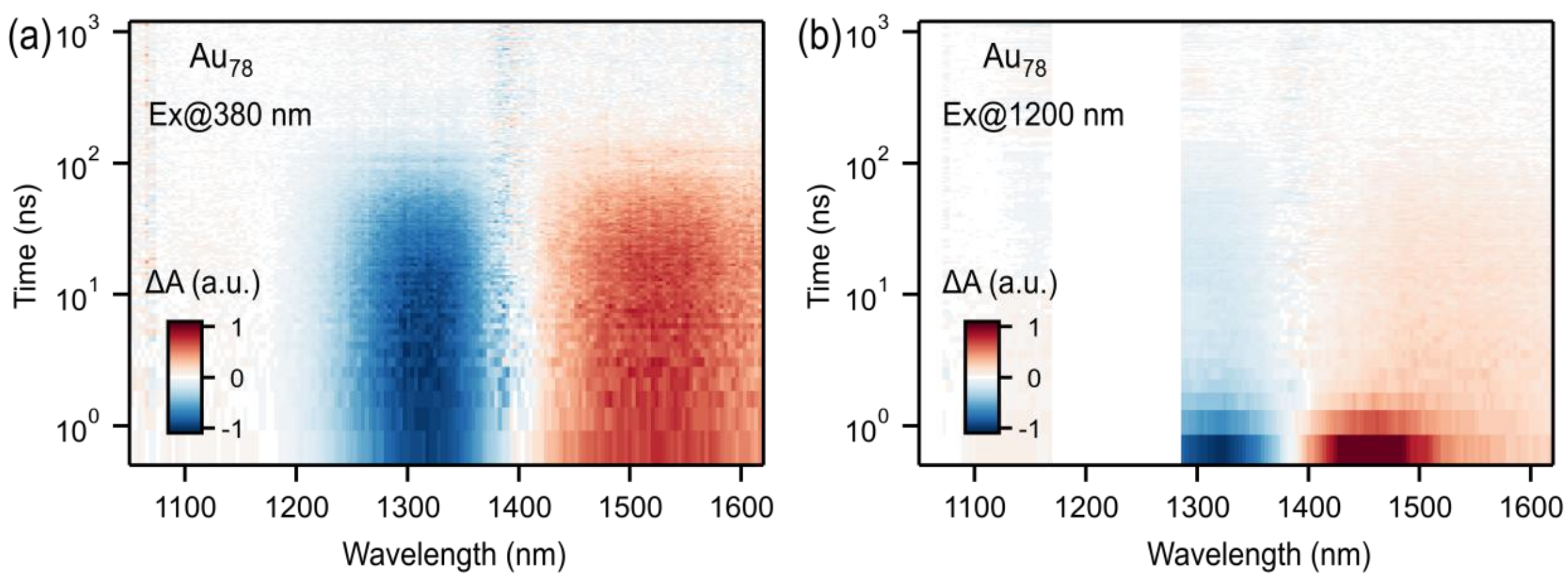

Figure S12. ns-TA of Au_{78} excited at 380 nm (a), and at 1200 nm (b). Note: we performed ns-TA by S_1 excitation at 1200 nm to obtain the GSB region (~1300 nm), and the coherent artifact around 1200 nm is removed in panel (b).

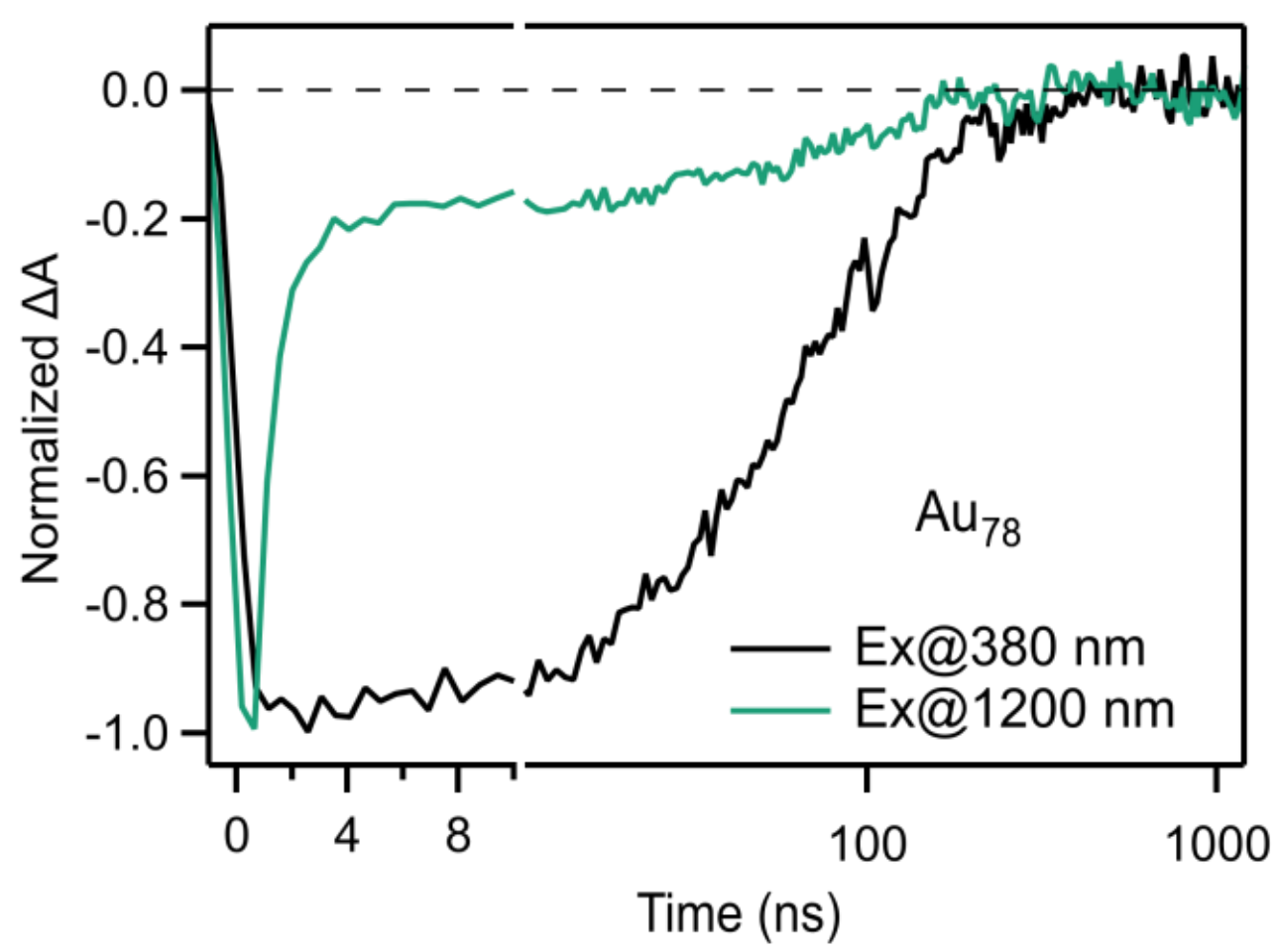

Figure S13. The extracted GSB decay (~1300 nm) in ns-TA of Au_{78} under excitation of 380 nm and 1200 nm, respectively.

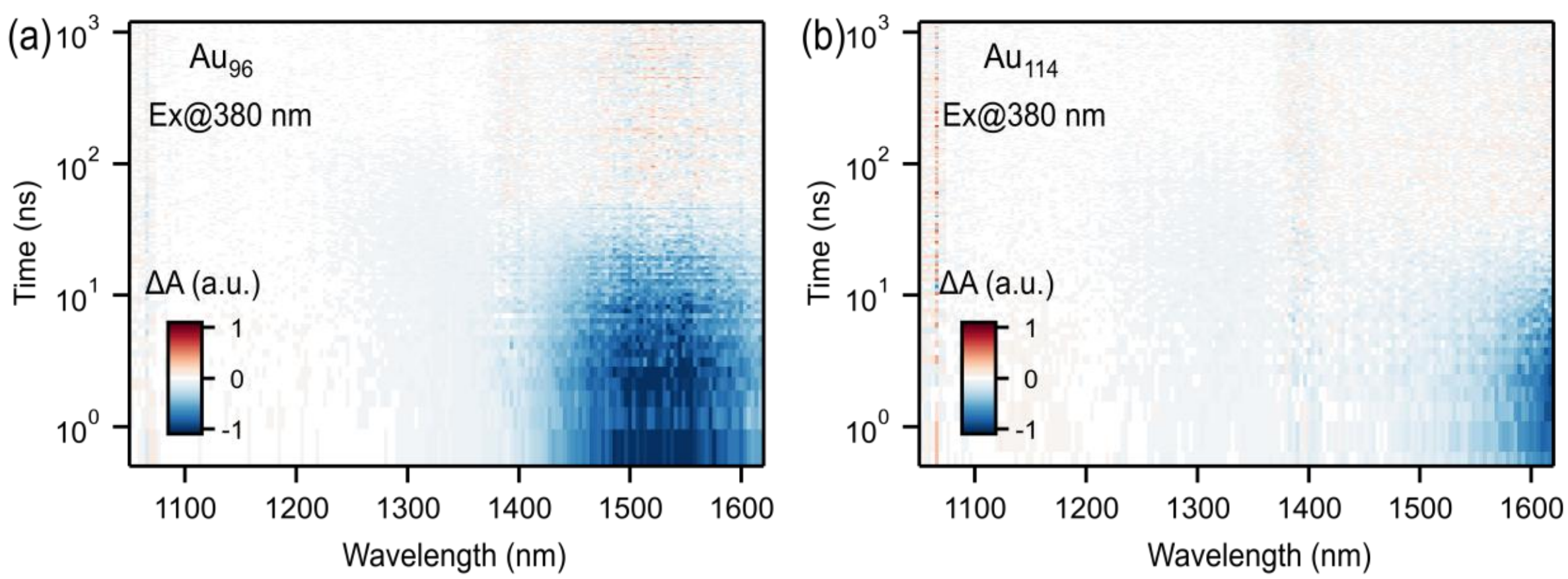

Figure S14. ns-TA of Au_{96} (a) and Au_{114} (b) excited at 380 nm. Note: the wavelength limit is ~1650 nm.

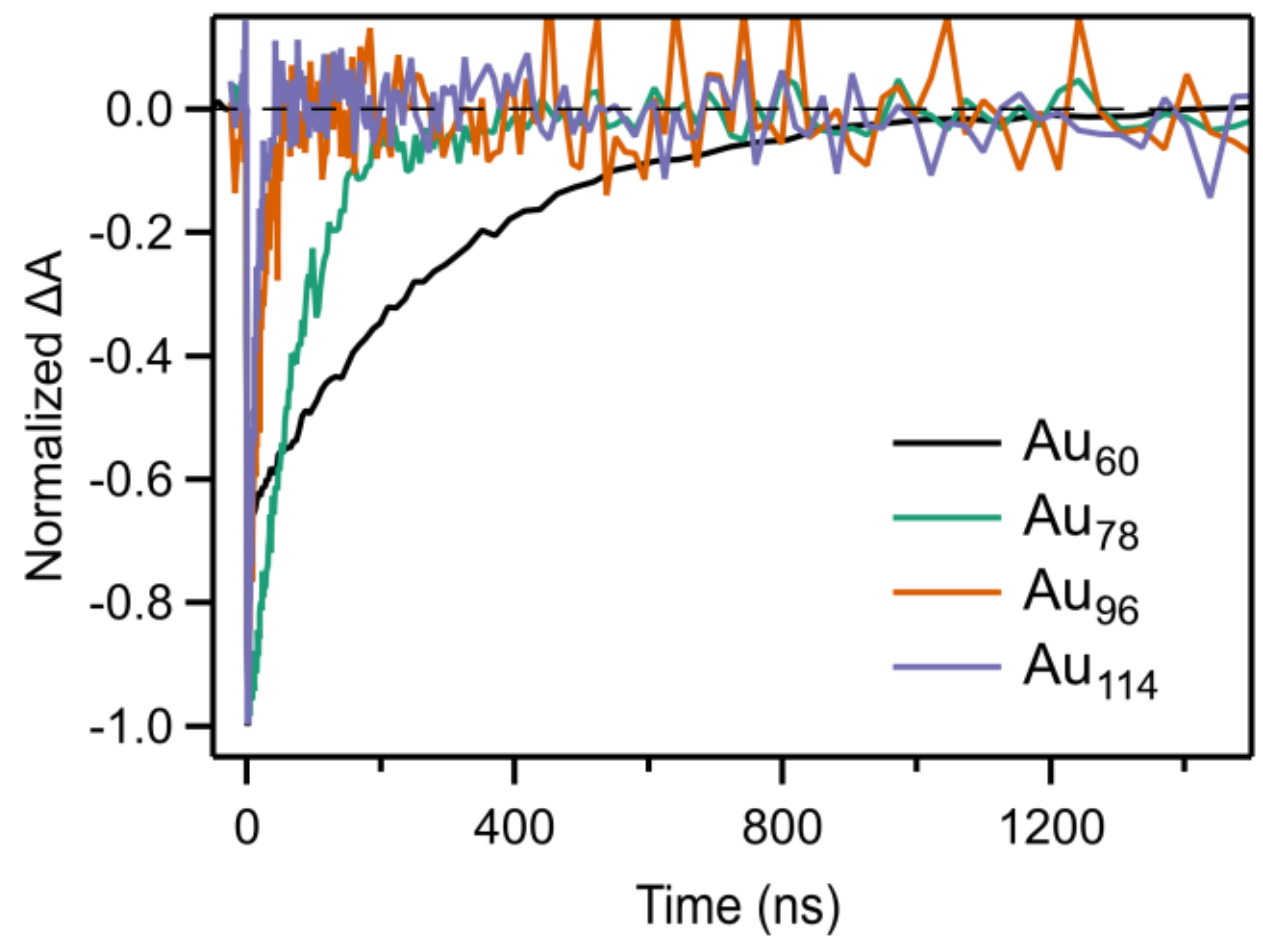

Figure S15. The bleach signal decay of the four Au QRs from ns-TA excited by 380 nm.

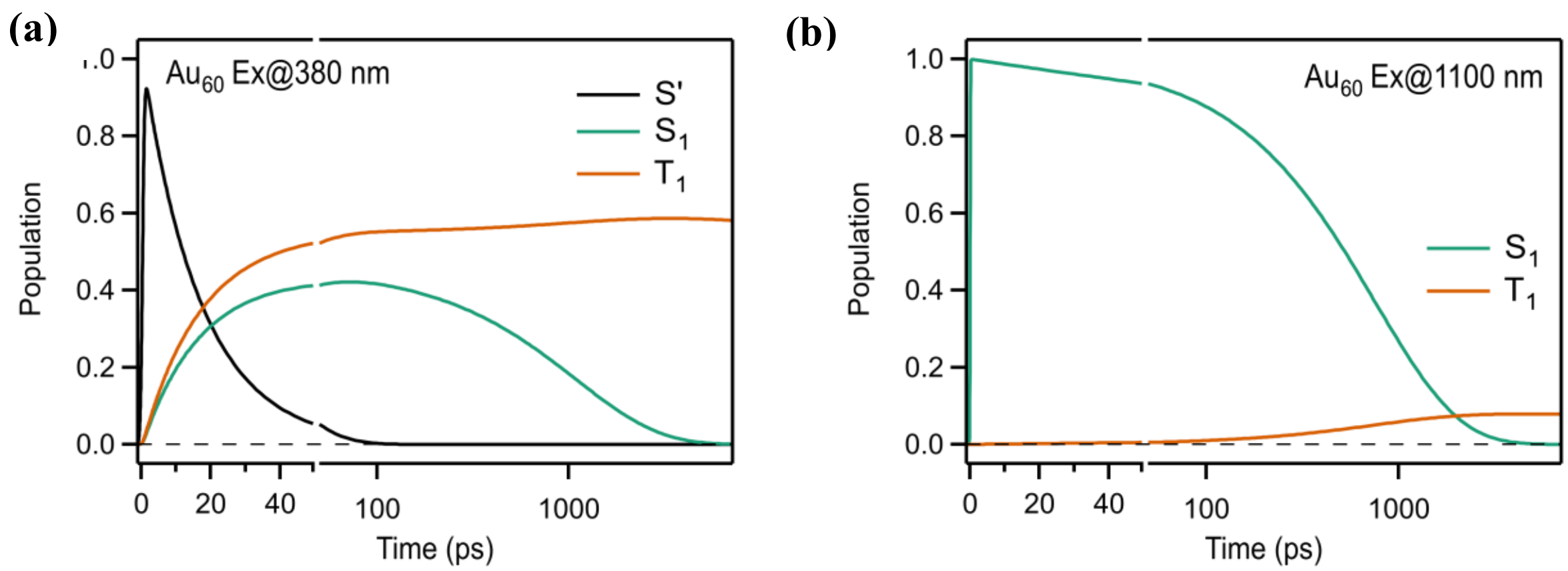

Figure S16. Excited states' populations of Au_{60} from the target analysis, (a) under 380 nm excitation, and (b) under 1100 nm excitation.

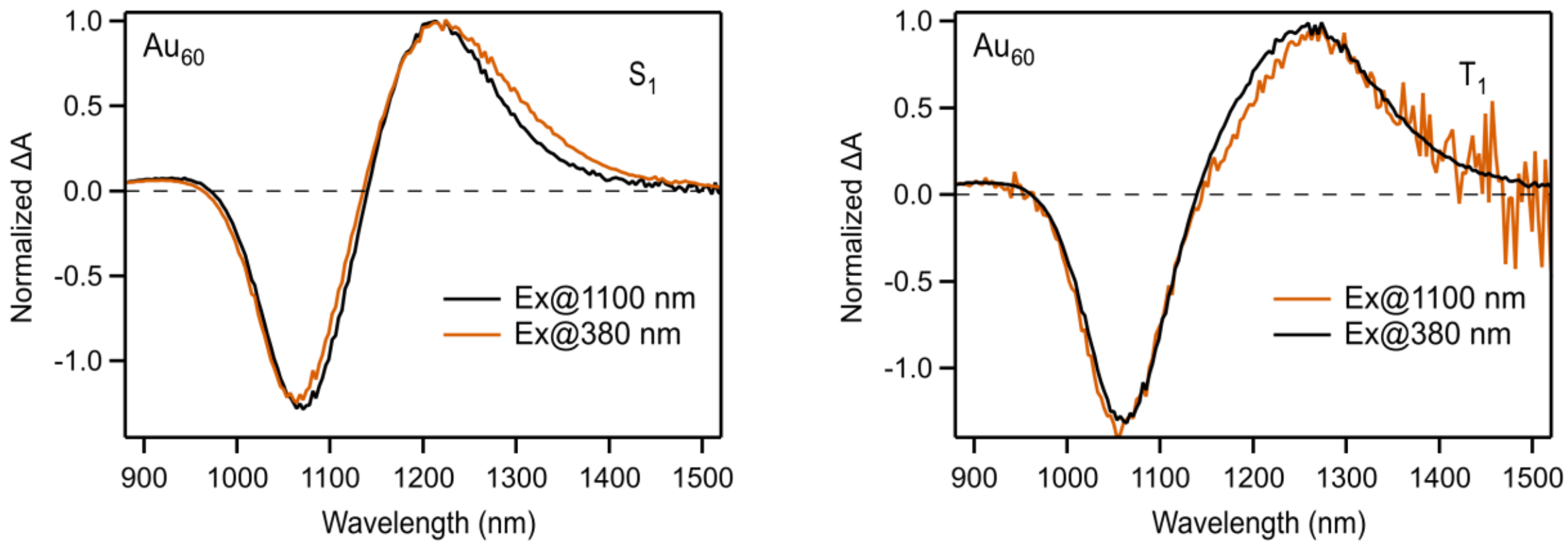

Figure S17. Spectral comparison of S_1 (left panel) and T_1 (right panel) at excitation of S_1 (1100 nm) and S_n (380 nm). Note: GSB at 1070 nm.

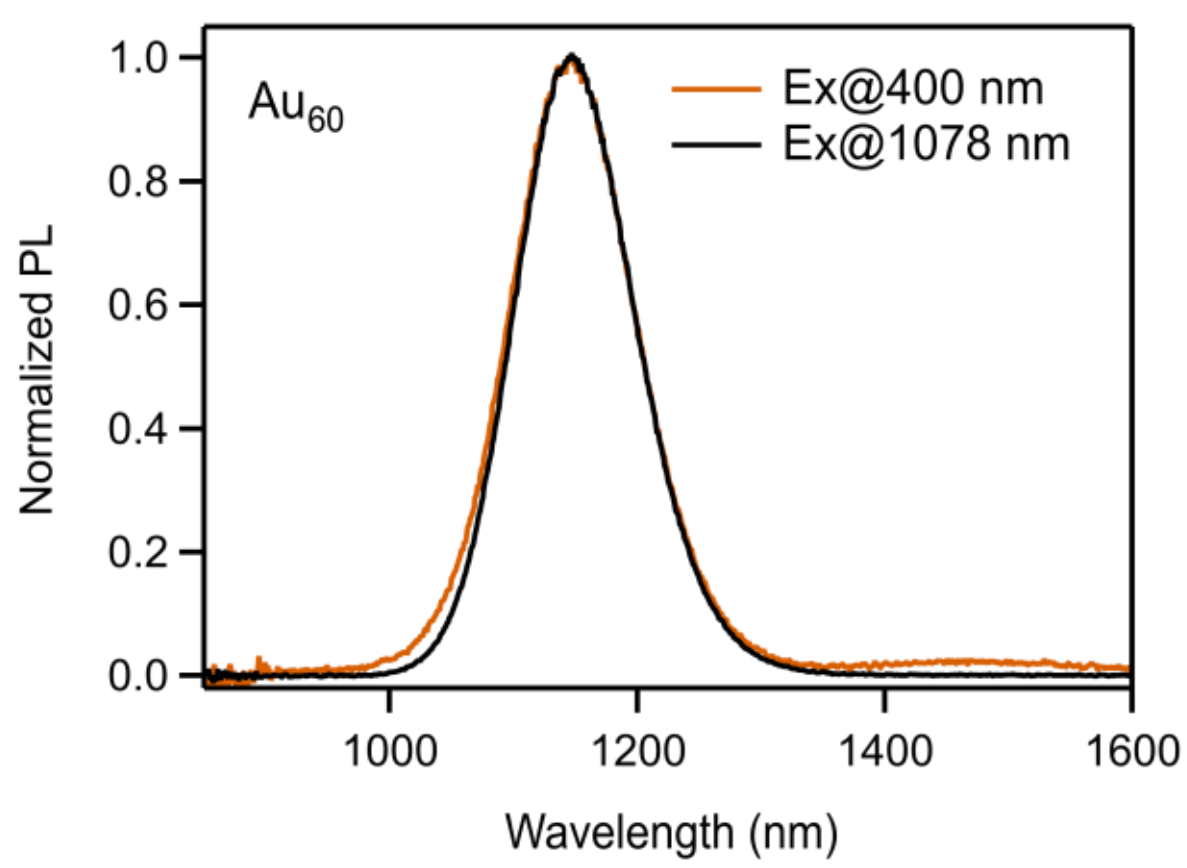

Figure S18. Comparison of PL spectra (normalized) of Au_{60} at excitation of S_1 (1078 nm) and S_n (400 nm), respectively.

Table S1. Target-fitting parameters for TA data of Au_{60} with different excitation wavelengths.

$\lambda_{excitation}$ (nm)	τ_1 $[S' \rightarrow S_1(b_1)/T(b_2)]$			τ_2 $[S_1 \rightarrow T_1(b_3)/S_0(b_4)]$			$\tau_3(T_1 \rightarrow S_0)$* (ns)
	τ_1 (ps)	b_1	b_2	τ_2 (ps)	b_3	b_4	
380	16.9	0.45	0.55	911	0.1	0.9	328
500	16.9	0.45	0.55	920	0.1	0.9	320
730	14.6	0.45	0.55	929	0.1	0.9	318
810	13.5	0.45	0.55	946	0.1	0.9	316
900	13.8	0.45	0.55	926	0.1	0.9	338
1000	--	--	--	926	0.1	0.9	325
1100	--	--	--	760	0.1	0.9	341

*Note: Triplet lifetime determined by ns-TA data.

Table S2. Target-fitting parameters for fs-TA of Au_{60} to Au_{114} QRs with corresponding S_1 excitation and S_n excitation (common 380 nm).

Nanorod Clusters	$\lambda_{excitation}$ (nm)	τ_1 $[S' \rightarrow S_1(b_1)/T(b_2)]$			τ_2 $[S_1 \rightarrow T_1(b_3)/S_0(b_4)]$			$\tau_3(T_1 \rightarrow S_0)^a$ (ns)
		τ_1 (ps)	b_1	b_2	τ_2 (ps)	b_3	b_4	
Au_{60}	380	16.9	0.45	0.55	911	0.1	0.9	328
	1100	--	--	--	760	0.1	0.9	341
Au_{78}	380	7.6	0.13	0.87	519	0.05	0.95	83
	1300	--	--	--	526	0.05	0.95	76^b
Au_{96}	380	19.6	0.21	0.79	76.4	0	1	21
	1550	--	--	--	76.1	0	1	--
Au_{114}	380	21	0.11	0.89	61.2	0	1	13
	1550	--	--	--	61.2	0	1	--

a. Triplet lifetime determined by ns-TA data.
b. Triplet lifetime of Au_{78} determined at excitation of 1200 nm.

Supporting references:

(1) Luo, L.; Liu, Z.; Kong, J.; Gianopoulos, C. G.; Coburn, I.; Kirschbaum, K.; Zhou, M.; Jin, R. Three-Atom-Wide Gold Quantum Rods with Periodic Elongation and Strongly Polarized Excitons. *Proc. Natl. Acad. Sci. U.S.A.* **2024**, *121* (10), e2318537121.

(2) Snellenburg, J. J.; Laptenok, S. P.; Seger, R.; Mullen, K. M.; Stokkum, I. H. M. van. Glotaran : A Java-Based Graphical User Interface for the R Package TIMP. *J. Stat. Soft.* **2012**, *49* (3), 1–22.

(3) van Stokkum, I. H. M.; Larsen, D. S.; van Grondelle, R. Global and Target Analysis of Time-Resolved Spectra. *Biochim. Biophys. Acta - Bioenerg.* **2004**, *1657* (2–3), 82–104.
